# A Multiple Step Active Stiffness Integration Scheme to Couple a Stochastic Cross-Bridge Model and Continuum Mechanics for Uses in Both Basic Research and Clinical Applications of Heart Simulation

**DOI:** 10.3389/fphys.2021.712816

**Published:** 2021-08-13

**Authors:** Kazunori Yoneda, Jun-ichi Okada, Masahiro Watanabe, Seiryo Sugiura, Toshiaki Hisada, Takumi Washio

**Affiliations:** ^1^Section Solutions Division, Healthcare Solutions Development Unit, Fujitsu Japan Ltd., Tokyo, Japan; ^2^UT-Heart Inc., Kashiwa, Japan; ^3^Future Center Initiative, University of Tokyo, Kashiwa, Japan

**Keywords:** heart simulation, Monte Carlo method, finite element method, excitation contraction coupling, multiple time step method, active stiffness, cross-bridge cycle

## Abstract

In a multiscale simulation of a beating heart, the very large difference in the time scales between rapid stochastic conformational changes of contractile proteins and deterministic macroscopic outcomes, such as the ventricular pressure and volume, have hampered the implementation of an efficient coupling algorithm for the two scales. Furthermore, the consideration of dynamic changes of muscle stiffness caused by the cross-bridge activity of motor proteins have not been well established in continuum mechanics. To overcome these issues, we propose a multiple time step scheme called the multiple step active stiffness integration scheme (MusAsi) for the coupling of Monte Carlo (MC) multiple steps and an implicit finite element (FE) time integration step. The method focuses on the active tension stiffness matrix, where the active tension derivatives concerning the current displacements in the FE model are correctly integrated into the total stiffness matrix to avoid instability. A sensitivity analysis of the number of samples used in the MC model and the combination of time step sizes confirmed the accuracy and robustness of MusAsi, and we concluded that the combination of a 1.25 ms FE time step and 0.005 ms MC multiple steps using a few hundred motor proteins in each finite element was appropriate in the tradeoff between accuracy and computational time. Furthermore, for a biventricular FE model consisting of 45,000 tetrahedral elements, one heartbeat could be computed within 1.5 h using 320 cores of a conventional parallel computer system. These results support the practicality of MusAsi for uses in both the basic research of the relationship between molecular mechanisms and cardiac outputs, and clinical applications of perioperative prediction.

## Introduction

**FIGURE 1:**
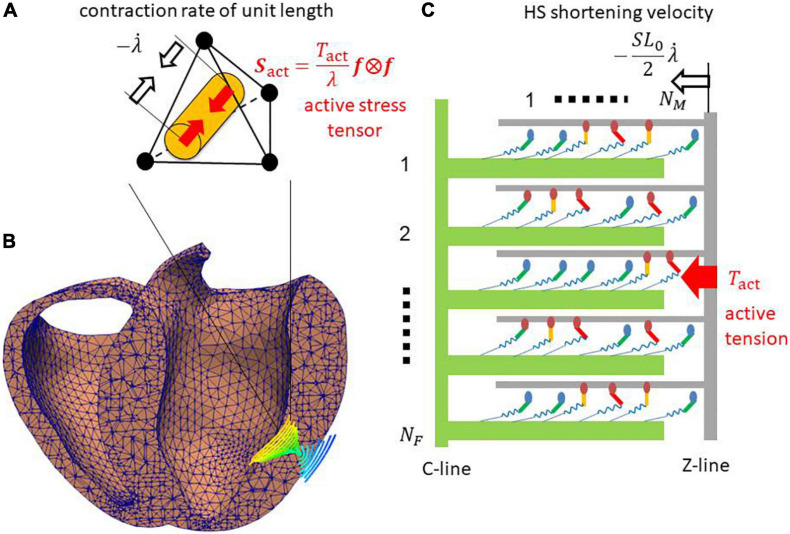
**(A)** Tetrahedral element in **(B)** the ventricle wall of the FE model. The cylinder in panel **(A)** indicates the fiber orientation ***f*** that is shown in panel **(B)** using the integral curves colored according to the longitudinal component of ***f*** (blue: –1, red: +1). **(C)** The half-sarcomere model was assigned for each element. The active tension *T*_*act*_ is given by summing the forces of the binding myosin molecules composed of the myosin head (ellipse), and the lever arm (bar) and rod (spring). In the half-sarcomere model, the C-line and thick filaments (green) are fixed. The Z-line and thin filament (gray) slide with the half-sarcomere (HS) shortening velocity $-S\,L_{0}\,\dot{\lambda }/2$, where the stretch rate $\dot{\lambda }$ along the fiber orientation ***f*** is obtained from the FE model. The active tension *T*_*act*_ is given by summing the forces generated by the binding myosin molecules, and it is used to define the macroscopic active stress tensor *S*_*act*_ that drives the heartbeat in the FE model.

**FIGURE 2:**
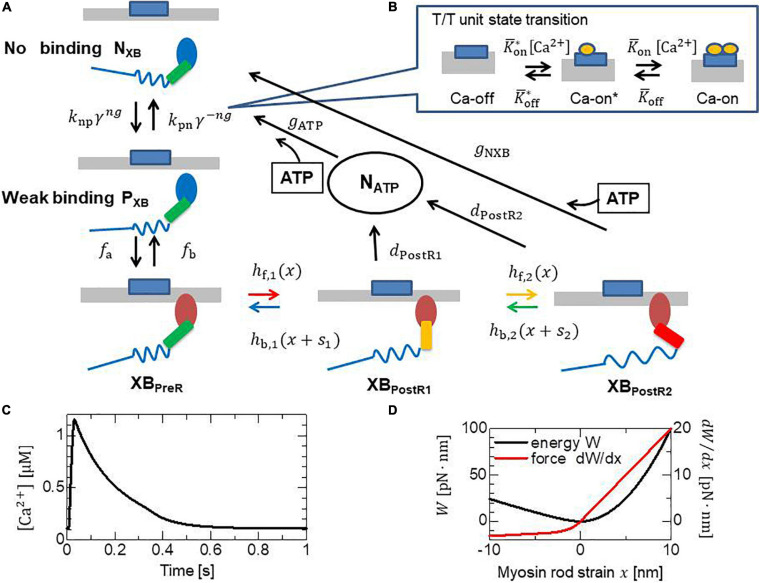
State transition Monte Carlo model of **(A)** the myosin molecule and **(B)** the T/T unit. The myosin molecules in either the N_*XB*_ or P_*XB*_ states are assumed to be detached. The rate constants in the T/T unit state transition are affected by the states of the myosin heads (MHs) below it. The rate constant factors *k*_*np*_ and *k*_*pn*_ between N_*XB*_ and P_*XB*_ are affected by the state of the T/T unit above it. *ng* is an integer that takes the values 0, 1, or 2, according to the number of neighboring MHs attached. γ = 40 was adopted to model the co-operativity of the MHs. The forward transitions from XB_*PreR*_ to XB_*PostRs*_ via XB_*PostR1*_ are called ‘power strokes,’ whereas the back transitions are called ‘reverse strokes.’ The MHs connected to the extremely strained myosin rods are detached to N_*ATP*_. **(C)** The typical transient of [Ca^2+^] applied to the T/T unit state transition in panel **(B)**. **(D)** The strain energy *W* of the myosin rod and the non-linear force *dW*/*dx*.

Demands for the prediction of outcomes from various types of operations are emerging in clinical problems of heart disease. At the present time, we can reconstruct a precise three-dimensional (3D) model from computed tomography or magnetic resonance imaging data for an individual patient, and use it for perioperative simulations to help doctors to choose the best among various possible operations. However, there are still some difficulties in the modeling of excitation-contraction coupling, even if we can precisely predict the excitation propagation from patient electrocardiogram (ECG) data (Okada et al., 2013). Such difficulties are because the macroscopic shortening in the fiber orientation is fed back to the extremely large stochastic combination consisting of various states of contractile proteins (Figure 1), and their stochastic responses depend on individual scenarios that include their neighbors (Figure 2). Although previous efforts to construct numerical models using a type of mean field approximation have successfully reproduced specific tissue-level phenomena (Hunter et al., 1998; Niederer et al., 2006; Negroni and Lascano, 2008; Rice et al., 2008; Guérin et al., 2011; Chapelle et al., 2012; Washio et al., 2012; Syomin and Tsaturyan, 2017; Regazzoni et al., 2018; Caruel et al., 2019), these models have not yet been fully exploited in real-life heart simulations. The uses of ordinary differential equation (ODE) models that adopt the phenomenological approximations of the force-pCa relationship and the force-velocity relationship have become mainstream instead (Smith et al., 2004; Kerckhoffs et al., 2007; Gurev et al., 2011; Shavik et al., 2017; Dabiri et al., 2019; Azzolin et al., 2020; Regazzoni et al., 2020). However, these approaches appear to have difficulties, particularly in reproducing the realistic relaxation phase that is important to ease the influx of blood from the atria to the ventricles.

Two major problems exist in directly coupling a stochastic molecular model and a living heart model: (i) the time scale difference between the two models; and (ii) the treatment of active stiffness in the heart model associated with the stochastic cross-bridge activity in the molecular model. Regarding the first problem, cross-bridge activity is typically modeled either using an ODE model or a Monte Carlo (MC) model that requires a small time step of microsecond order, whereas millisecond order is appropriate for the heart model discretized by the finite element method (FEM) in terms of the computational load and communication overhead. Regarding the second problem, an implicit method is typically applied for the FE model because of the strong anisotropic and volumetric stiffness of living tissue. When a muscle is excited, active stiffness associated with cross-bridge activity is generated in the fiber orientation (Figure 1B). This active stiffness is much greater than passive stiffness, with the exception of the volumetric stiffness of the incompressibility. Thus, if the prescribed active tensions computed in the cross-bridge model are explicitly applied to the FE model, active stiffness is not taken into account in the total stiffness matrix of the Newton iteration, which causes some problems of either convergence or accuracy. Such a problem was analyzed by Regazzoni and Quarteroni (2021), and the instability was fixed by introducing an appropriate active stiffness. However, their study was limited to some phenomenological ODE models that cannot reproduce a spontaneous oscillation (SPOC) (Ishiwata et al., 2010; Kagemoto et al., 2015) in low Ca^2+^ concentrations, as Regazzoni et al. (2021) remarked. By contrast, we successfully reproduced a SPOC (Washio et al., 2019; Shintani et al., 2020) using our MC cross-bridge model (Figure 2), and we addressed the similarity between the rapid lengthening of sarcomeres in the SPOC and the quick relaxation of cardiac muscle at early diastole in the cardiac cycle. Therefore, in this study, we focus on the stability in the direct coupling of the MC and FE models. The similarities of the cross-bridge dynamics in the relaxation phases between the SPOC and the biventricular FE simulations demonstrate the usefulness of our scheme both in areas of basic research and clinical applications.

## Materials and Methods

### Coupling of the MC Model and the FEM Model

**FIGURE 3:**
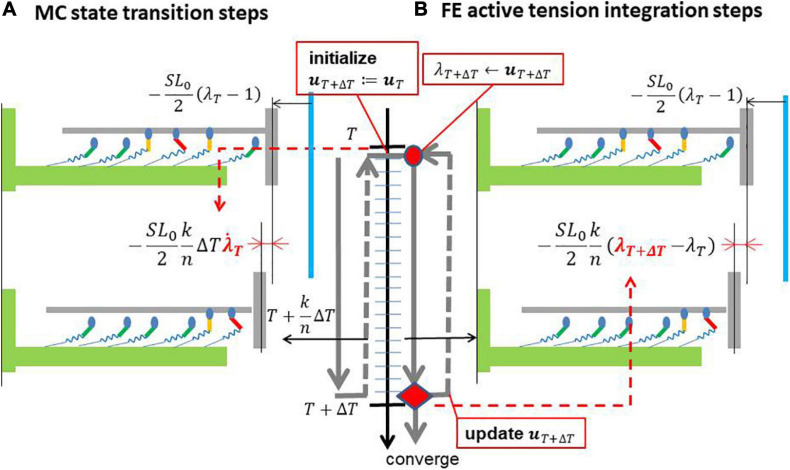
Difference between the sliding distances of the thin filament in **(A)** the MC state transition steps and **(B)** the FE active tension integration steps. An FE time interval [*T*,*T* + Δ*T*] is divided into *n* MC multiple steps (*k* = 1,⋯,*n*). The vertical blue line is the position of the Z-line under the unloaded condition. The sliding distances in [*T*,*T* + Δ*T*] are extrapolated from the sliding velocity $-{\dot{\lambda }}_{T}\cdot S\,L_{0}/2$ at *T* in the MC steps, whereas they are interpolated from the stretches λ_*T*_ and λ_*T+ΔT*_ at *T* and *T* + Δ*T* in the active tension integration steps, respectively. The gray arrows around the time axis indicate the flow of the computational process. The MC state transition steps are performed only once from *T* to *T* + Δ*T*, whereas the FE active tension integration steps are iterated until the convergence of Newton iterations, where λ_*T+ΔT*_ is computed from the updated displacement **u**_*T* + Δ*T*_. Note that **u**_*T* + Δ*T*_ is initialized with ***u***_*T*_ at the beginning of the Newton iterations in this study.

In this study, we apply a multiple time step approach in which about 100 time steps of the MC model are performed within a single time step of the FE model to reduce the computational load and communication overhead (Figure 3). In our approach, to update the variables in both the MC and FE models from the FE time step at *T* to the next time step at *T* + Δ*T*, first, the stretch λ_*T*_ and stretch rate ${\dot{\lambda }}_{T}$ in the fiber orientation of the FE model are used as the initial half-sarcomere length (HSL) λ_*T*_⋅*SL*_0_/2 at *T* and its shorting velocity $-{\dot{\lambda }}_{T}\cdot S\,L_{0}/2$ in the time interval [*T*,*T*Δ*T*] of the half-sarcomere model (Figure 3A), where *SL*_0_ is the sarcomere length under the unloaded condition. This filament sliding information is used to calculate the myosin rod strains that are referenced to compute the state transitions of binding myosin molecules. Then, the active tension *T*_*act,[T,T+ΔT]*_ and associated stiffness ∂⁡*T*_*act*,[*T*,*T* + Δ*T*]_/∂⁡λ_*T* + Δ*T*_ are computed iteratively in Newton iterations by summing the individual contributions of binding myosin molecules. The myosin rod strains are recomputed using the interpolated stretches between λ_*T*_ and λ_*T* + Δ*T*_ in the time interval [*T*,*T* + Δ*T*], whereas the myosin molecule states already computed in the MC steps are fixed (Figure 3B) for convergence in the Newton iterations. Thus, the stretch λ_*T* + Δ*T*_ is implicitly integrated into the active tension, which results in the appropriate evaluation of active stiffness in the FE model and the stability of the Newton iterations. Hereafter, we call our approach the “Multiple step Active stiffness integration” (MusAsi) scheme.

In our previous works (Washio et al., 2013, 2016), the active tension *T*_*act,[T,T+ΔT]*_ was implicitly computed by assuming the shorting velocity $-{\dot{\lambda }}_{T+\Delta \,T}\cdot S\,L_{0}/2$ of the half-sarcomere model in the time interval [*T*,*T* + Δ*T*], whereas in the MusAsi scheme, it is computed by assuming −(λ_*T* + Δ*T*_−λ_*T*_)⋅*SL*_0_/2Δ*T*. Although both approaches produce almost the same result, the previous approach is based on the velocities of the continuum, which result in the inconsistent stiffness of the active tension. By contrast, active stiffness in the MusAsi scheme is proportional to the total stiffness of the binding myosin molecules in the half-sarcomere model, thus the interpretation is consistent with our intuition. In the following, we introduce the MC model applied in this study and the details of the MusAsi scheme.

### MC Cross-Bridge Model

We briefly present an overview of our MC cross-bridge model (Washio et al., 2016) used in this study. We provide the details in Supplementary Material 1. A myosin molecule in our cross-bridge model has three non-binding states (N_XB_, P_*XB*_, and N_ATP_) and three strong binding states (XB_PreR_, XB_PostR1_, and XB_PostR2_) (Figure 2A). Ca^2+^-sensitivity is reproduced using the state transitions in the troponin/tropomyosin (T/T) units on the thin filament (Figure 2B). The coefficients *k*_*np*_ and *k*_*pn*_ in the rate constants between the non-binding state N_XB_ and the weakly binding state P_XB_ are changed according to the state of the T/T unit above the myosin molecule. Co-operativity in the nearest neighbor interactions is incorporated with the factors γ^ng^ and γ−^*ng*^ to reproduce the force-pCa^2+^ relationship (Rice et al., 2003), where γ= 40 is used, and *ng* = 0, 1, or 2 is the number of neighboring myosin molecules either in the weakly binding (P_XB_) or strong binding (XB_PreR_, XB_PostR1_, and XB_PostR2_) states. We assume that one real thin filament in the 3D arrangement corresponds to two thin filaments in our half-sarcomere model. This is because we assume that co-operative behavior exists along the tropomyosin and tropomyosin molecules wrapped around the thin filament in a double spiral manner, and only one of the spirals is considered in our half-sarcomere model. As shown in the “Results” section, this co-operative mechanism contributes to almost completely removing the population of binding states in the diastolic phase in which nearly 10% of the peak Ca^2+^ concentration remains (Figure 2C).

Contraction force is generated by the power stroke transitions in which the strain of the myosin rod increases by *s*_1_ and *s*_2_ in the first and second strokes, respectively. In our model, the rate constants of the power and reverse strokes are given by functions of the rod strain *x* (the displacement from the unloaded state) so that the Boltzmann equilibrium condition is fulfilled:

(1) $$\frac{h_{f,i}\,\left(x\right)}{h_{b,i}\,\left(x+s_{i}\right)}=\text{exp}\,\left(\frac{-W\,\left(x\right)-E_{i-1}+W\,\left(x+s_{i}\right)+E_{i}}{k_{B}\,T}\right),$$

where *E*_*i–1*_ and *E*_*i*_ are the free energies before and after the power stroke under the unloaded condition, respectively. *W* is the strain energy of the myosin rod (Figure 2D). With the power stroke, the free energy decrease *E*_*i*−1_−*E*_*i*_ is transferred to the increase of strain energy *W*(*x* + *s*_*i*_)−*W*(*x*). The strain energy is used for the external work via the half-sarcomere shortening that corresponds to muscle shortening in the fiber direction. In this study, the individual rate constants for the power and reverse strokes are determined by

$$\left\{ \begin{array}{c} h_{f,i}\,\left(x\right)=h_{i}\,\text{exp}\,\left(-\frac{E_{i-1}-W\,\left(x+s_{i}\right)-E_{i}}{k_{B}\,T}\right)\;\left(2\right)\\ h_{b,i}\left(x+s_{i}\right)=h_{i}\text{exp}\left(-\frac{W\,\left(x\right)}{k_{B}\,T}\right).\;\left(3\right) \end{array}\right..$$

To achieve stable MC steps, if either *h*_*f,i*_ or *h*_*b,i*_ exceeds the maximum rate *r*_max_ = 100,000 [1/*s*], it is replaced by *r*_*max*_ and the other parameter is modified so that Eq. 1 is fulfilled.

### MusAsi Scheme

For the MC model, the time step size Δ*t* must be chosen so that it is sufficiently smaller than the reciprocals of the rate constants. In our case, the choice Δ*t*~5μs is appropriate. By contrast, the FE time step size Δ*T*∼1*ms* is sufficient to catch the time transients of macroscopic variables, such as the ventricular cavity volume and pressure. Because the linear solution, which requires many communications among processes, must be performed in each Newton iteration, a FE time step size Δ*T* that is much larger than Δ*t*∼5μs is desirable. This leads to the use of an approach in which multiple MC steps are performed in a single FE step.

The feedback to the state transitions in the MC model from the dynamics of the FE model is given by the stretch in the fiber orientation ***f***. In the FE model, the stretch and stretch rate are given by

(4) λ=||Ff||

(5) $$\dot{\lambda }=\frac{1}{\lambda }\,\left(\text{Ḟ}\,\text{f}\right)\cdot \left(\mathrm{Ff}\right)$$

where **F** = **I** + ∂⁡**u**/∂⁡**X** is the deformation gradient tensor defined for the displacements **u** = **u**(*T*,**X**) from the unloaded configuration. The macroscopic information of the stretch is provided at the two ends of the time interval at *T* and *T* + Δ*T*. The information at *T* + Δ*T* is determined when the macroscopic displacement **u** = **u**(*T* + Δ*T*,**X**) is determined, which can be computed only if the active stress during [*T*,*T* + Δ*T*] is provided. Thus, the state transitions of myosin molecules in MC model must be computed before the FE step from *T* to *T* + Δ*T*. Hereafter, we denote the time using a subscript, if necessary, for example, λ_*T*_ and ${\dot{\lambda }}_{T}$. A simple approach to perform the MC steps with the time step size Δ*t* = Δ*T*/*n* is to define the stretch $\tilde{\lambda }$ at the *k*-th step as

(6) $${\tilde{\lambda }}_{T+k\,\Delta \,t}\equiv \lambda_{T}+k\,\Delta \,t\,{\dot{\lambda }}_{T}.$$

In this case, the rod strain ${\tilde{x}}_{i\,j}$ (displacement from the unloaded position) of the (*i,j*)-th myosin molecule is given by the following if it is in the binding state:

(7) $${\tilde{x}}_{i\,j,T+k\,\Delta \,t}=x_{A,i\,j,T+k\,\Delta \,t}+s_{i\,j,T+k\,\Delta \,t}+\frac{S\,L_{0}}{2}\,\left({\tilde{\lambda }}_{T+k\,\Delta \,t}-{\tilde{\lambda }}_{A,i\,j,T+k\,\Delta \,t}\right)$$

where the integers *i* = 1,⋯,*N*_*M*_ and *j* = 1,⋯,*N*_*F*_ denote the index of a myosin molecule in a filament and the index of a thin filament in the half-sarcomere model imbedded in a single finite element in the FE model (Figure 1), respectively. The variables *x*_*A,ij*_ and ${\tilde{\lambda }}_{A,i\,j}$ are the initial strain and the stretch at the most recent attachment (the transition from P_*XB*_ to XB_*PreR*_), respectively. The initial strain *x*_*A,ij*_ is yielded probabilistically from the Boltzmann distribution exp(−*W*(*x*)/*k*_*B*_*T*). The variable *s*_*ij*_ = 0,*s*_1_ or *s*_1_ + *s*_2_ is the power stroke distance. The third term on the right-hand side is the sliding distance between the thin filament and the thick filament after the attachment. The constant *SL*_0_ is the sarcomere length under the unloaded condition. In the MC computation, a state transition of a binding myosin molecule *ij* at *k*-th step is computed based on the rate constant determined by the rod strain ${\tilde{x}}_{i\,j,T+k\,\Delta \,t}$.

Once the state transitions in the MC model in the time interval [*T*,*T* + Δ*T*] are computed, the active tension *T*_*act,[T,T+ΔT]*_ of the FE model is calculated by summing all the forces produced by individual myosin molecules so that the impulses of both scales are the same:

(8) $$T_{a\,c\,t,\left[T,T+\Delta \,T\right]}=\frac{2\cdot R_{S}}{S\,A_{0}\cdot n_{F}\cdot n}\,\sum_{j=1}^{N_{F}}\sum_{i=1}^{N_{M}}\sum_{k=1}^{n}\delta_{A,i\,j,T+\kappa \,\Delta \,t}\,\frac{d\,W}{d\,x}\,\left(x_{i\,j,T+k\,\Delta \,t}\right),$$

where *SA*_0_ and *R_S_* are the cross-sectional area per thin filament and the sarcomere volume ratio under the unloaded condition, respectively. The numerator is multiplied by a factor 2 because we consider a half the myosin molecules (*N*_*M*_ = 38), which are accessible along a single spiral in the thin filament for the purpose of co-operative attach-detach control along the tropomyosin. The variables {δ_*A*,*ij*,*T* + κΔ*t*_ = 0: non-binding, =1: binding} are obtained from the MC model. Regarding the forces generated by the individual myosin molecules, if we define the rod strain as $x_{i\,j,T+k\,\Delta \,t}\equiv {\tilde{x}}_{i\,j,T+k\,\Delta \,t}$, the active tension *T*_*act,[T,T+ΔT]*_ is determined regardless of the stretch λ_*T* + Δ*T*_ at *T* + Δ*T*. Therefore, the active tension stiffness *dT*_*act*,[*T*,*T* + Δ*T*]_/*d*λ_*T* + Δ*T*_ associated with the binding myosin molecules in the half-sarcomere model is not taken into account in the total stiffness matrix used in the FE Newton iterations. This approach, which considers the active tension explicitly, results in the instability of the numerical solution, as seen in the “Results” section. Therefore, in the MusAsi scheme, the rod strains {*x*_*ij*,*T* + *k*Δ*t*_} used to determine the active tension are given by

(9) $$x_{i\,j,T+k\,\Delta \,t}\equiv x_{A,i\,j,T+k\,\Delta \,t}+s_{i\,j,T+k\,\Delta \,t}+\frac{S\,L_{0}}{2}\,\left(\lambda_{T+k\,\Delta \,t}-\lambda_{A,i\,j,T+k\,\Delta \,t}\right),$$

where the variables {*x*_*A*,*ij*,*T* + *k*Δ*t*_*s*_*ij*,*T* + *k*Δ*t*_} produced in the MC steps *k* = 1,⋯,*n* are used, whereas the current stretch λ_*T* + *k*Δ*t*_ and the stretch λ_*A*,*ij*,*T* + *k*Δ*t*_ at the most recent attachment are sequentially redefined from the stretch λ_*T* + Δ*T*_ at the time step *T* + Δ*T* as follows:

(10) $$\lambda_{T+k\,\Delta \,t}\equiv \lambda_{T}+\frac{k}{n}\,\left(\lambda_{T+\Delta \,T}-\lambda_{T}\right)$$

(11) $$\lambda_{A,i\,j,T+k\,\Delta \,t}\,\left\{ \begin{array}{c} \lambda_{T+k\,\Delta \,T},\delta_{A,i\,j,T+\kappa \,\Delta \,t}=1\,a\,n\,d\,\delta_{A,i\,j,T+\left(k-1\right)\,\Delta \,t}=0\\ \lambda_{A,i\,j,T+\left(k-1\right)\,\Delta \,t},o\,t\,h\,e\,r\,w\,i\,s\,e. \end{array}\right.$$

By including λ_*T* + Δ*T*_ in the definition of rod strain {*x*_*ij*,*T* + *k*Δ*t*_}, the stiffness associated with cross-bridge activity at *T* + Δ*T* is properly represented as

(12) $$\frac{\partial T_{a\,c\,t,\left[T,T+\Delta \,T\right]}}{\partial \lambda_{T+\Delta \,T}}=\frac{2}{S\,A_{0}\cdot N_{F}\cdot n}\,\sum_{j=1}^{N_{F}}\sum_{i=1}^{N_{M}}\sum_{k=1}^{n}\delta_{A,i\,j,T+\kappa \,\Delta \,t}\,\frac{d^{2}\,W}{d\,x^{2}}\,\left(x_{i\,j,T+k\,\Delta \,t}\right)\,\frac{\partial x_{i\,j,T+k\,\Delta \,t}}{\partial \lambda_{T+\Delta \,T}}$$

where the derivative with respect to λ_*T* + Δ*T*_ is given by

(13) $$\frac{\partial x_{i\,j,T+k\,\Delta \,t}}{\partial \lambda_{T+\Delta \,T}}=\frac{S\,L_{0}}{2}\,\left(\frac{\partial \lambda_{T+k\,\Delta \,t}}{\partial \lambda_{T+\Delta \,T}}-\frac{\partial \lambda_{A,i\,j,T+k\,\Delta \,t}}{\partial \lambda_{T+\Delta \,T}}\right)$$

with

(14) $$\frac{\partial \lambda_{T+k\,\Delta \,t}}{\partial \lambda_{T+\Delta \,T}}=\frac{k}{n}$$

and

(15) $$\frac{\partial \lambda_{A,i\,j,T+k\,\Delta \,t}}{\partial \lambda_{T+\Delta \,T}}=\left\{ \begin{array}{c} \frac{k}{n},\delta_{A,i\,j,T+\kappa \,\Delta \,t}=1\,a\,n\,d\,\delta_{A,i\,j,T+\left(k-1\right)\,\Delta \,t}=0\\ \frac{\partial \lambda_{A,i\,j,T+\left(k-1\right)\,\Delta \,t}}{\partial \lambda_{T+\Delta \,T}},o\,t\,h\,e\,r\,w\,i\,s\,e \end{array}\right.$$

starting from $\frac{\partial \lambda_{A,i\,j,T+k\,\Delta \,t}}{\partial \lambda_{T+\Delta \,T}}=0$ at *k=0*. From Eqs 14, 15, we obtain

(16) $$\frac{\partial x_{i\,j,T+k\,\Delta \,t}}{\partial \lambda_{T+\Delta \,T}}\geq 0.$$

Therefore, the stiffness ∂⁡*T*_*act*,[*T*,*T* + Δ*T*]_/∂⁡λ_*T* + Δ*T*_ is always non-negative, provided the potential *W* is convex downward (*d*^2^*W*/*dx*^2^≥0).

### Active Stress and Stiffness in the FE Model

The infinitesimal external work per unit volume required to make an infinitesimal increment of stretch δλ against the active tension *T*_*act*_ is given by

(17) $$\delta \,W_{e\,x\,t}=T_{a\,c\,t}\,\delta \,\lambda .$$

From the relationship between the stretch λ and the Green–Lagrange strain tensor **E** = (**F^T^F**−**I**)/2, we obtain

(18) $$\text{f}⊗\text{f}:\text{E}=\frac{1}{2}\,\left(\lambda^{2}-1\right).$$

Thus, the infinitesimal increment of stretch is represented by

(19) $$\delta \,\lambda =\frac{1}{\lambda }\,\text{f}⊗\text{f}:\delta \,\text{E}.$$

Therefore, if we define the second Kirchhoff active stress tensor as

(20) $${\text{S}}_{a\,c\,t}=\frac{T_{a\,c\,t}}{\lambda }\,\text{f}⊗\text{f},$$

the infinitesimal work is represented by δ*W*_*act*_ = **S**_*act*_:δ**E**.

The derivative of the infinitesimal work is given by

(21) $$\delta^{2}\,W_{a\,c\,t}=\frac{\partial T_{a\,c\,t}}{\partial \lambda }\,\delta \,\lambda^{2}+T_{a\,c\,t}\,\delta^{2}\,\lambda .$$

If we assume *T*_*act*_≡*T*_*act*,[*T*,*T* + Δ*T*]_ and λ≡λ_*T* + Δ*T*_, the first term on the right-hand side is non-negative from Eq. 16. The second term is also non-negative, provided the active tension *T*_*act*_ is non-negative because the Hessian of λ is represented as

(22) $$\delta^{2}\lambda =\frac{1}{\lambda }\left(\text{f}⊗\text{f}:\delta^{2}\text{E}-\frac{1}{\lambda^{2}}{\left(\text{f}⊗\text{f}:\delta \text{E}\right)}^{2}\right)=\frac{1}{\lambda }\,\left(\left(\delta \,\text{Ff}\right)\cdot \left(\delta \,\text{Ff}\right)-{\left(\text{a}\cdot \left(\delta \,\text{Ff}\right)\right)}^{2}\right),$$

with the normal fiber orientation vector in the current coordinate **a** = **Ff**/λ. Therefore, δ^2^*W*_*act*_ is non-negative, provided the active tension *T*_*act*_ is non-negative. This guarantees the stability of the MusAsi scheme during the contraction phase. Some cases of negative active tension *T*_*act*_ < 0may exist. In this case, the negative stiffness appears in the subspace of variations of deformation gradients δ**F** that satisfy **a**⋅(δ**Ff**) = 0. However, because the negative tension is typically small compared with the positive term from the mass, viscosity, and passive stiffness, stability is guaranteed for an appropriately small time step size Δ*T*∼1ms, in our experience. When some impaired cross-bridge models were tested, we also observed that the deletion of the second term on the right-hand side of Eq. 21 from the stiffness matrix for negative active tensions (*T*_*act*_ < 0) further stabilized the convergence of Newton iterations in the MusAsi scheme. In our previous approach (Washio et al., 2016), because $\lambda_{T+k\,\Delta \,t}\equiv \lambda_{T}+k\,\Delta \,t\,{\dot{\lambda }}_{T+\Delta \,T}$ was used instead of Eq. 10, the first term in Eq. 21 was replaced with $\left(\partial T_{a\,c\,t}/\partial \dot{\lambda }\right)\,\delta \,\lambda \,\partial \dot{\lambda }$. Therefore, the interpretation of this term as the stiffness was difficult, although we did not have any convergence difficulty.

### Newton Iterations for the FE Model

In this study, the FE biventricular model was connected with the systemic and pulmonary circulation models, and the transfer of blood volume using these circulation models was described only by the volumetric changes of ventricular cavities. Thus, the combined system of equations for the FE model is given by the following six formulas for the biventricular FE (Eqs 23–26), systemic circulation (Eq. 27), and pulmonary circulation models (Eq. 28):

$$\left\{ \begin{array}{l} \;\int_{\Omega }^{}\text{δ}u\cdot \rho ű\;d\Omega +\int_{\Omega }^{}\text{δ}E:(S-pC^{-1})d\Omega \\ \begin{array}{ll} -P_{L}\int_{\Gamma_{\text{L}}}^{}\text{δ}u\cdot n\;d\Gamma_{L}-P_{R}\int_{\Gamma_{R}}^{}u\cdot n\;d\Gamma_{R}=0 & \left(23\right)\\ \int_{\Omega }^{}\text{δ}p\left((J-1)+\frac{p}{k}\right)d\Omega =0 & \left(24\right)\\ \int_{\Gamma_{L}}^{}\dot{u}\cdot n\;d\Gamma_{L}-(F_{MI}-F_{AO})=0 & \left(25\right)\\ \int_{\Gamma_{R}}^{}\dot{u}\cdot n\;d\Gamma_{R}-(F_{TR}-F_{PA})=0 & \left(26\right)\\ C_{S}(P_{L},F_{AO},\;Q_{S},\;F_{TR},\;P_{R})=0 & \left(27\right)\\ C_{P}(P_{R},F_{PA},\;Q_{P},\;F_{MI},\;P_{L})=0, & \left(28\right) \end{array} \end{array}\right.$$

where *J* = *det***F** is the Jacobian, *p* is the hydrostatic pressure in the ventricular walls, **C** = **F^T^F** is the right Cauchy-Green deformation tensor, κ is the bulk modulus, and *P_L_* = *LVP* and *P_R_* = *RVP* are blood pressure in the left and right ventricular cavities, respectively. is the muscle domain in the reference configuration, whereas Γ_*L*_ and Γ_*R*_ are the blood–muscle interfaces of the left and right ventricles, respectively, in the configuration at time *T*, and ***n*** is the normal unit vector directed from the cavity to the muscle at these surfaces. The Dirichlet boundary condition **u**_*T*_(**X**)≡0 is imposed on the boundary nodes around the valve rings. The second Piola–Kirchhoff stress tensor ***S*** consists of the active, passive, and viscous stresses:

(29) $$\text{S}\equiv {\text{S}}_{a\,c\,t}+{\text{S}}_{p\,a\,s}+{\text{S}}_{v\,i\,s}$$

where **S**_*act*_ is given by Eq. 20, and **S**_*pas*_ and **S**_*vis*_ are the passive and viscous stresses, respectively. The ventricle blood pressures *P_L_* and *P_R_* are determined through their interactions with the circulatory system of the body. *F*_*MI*_, *F*_*AO*_, *F*_*TR*_, and *F*_*PA*_ are the flow rates through the mitral, aortic, tricuspid, and pulmonary valves, respectively. **Q**_*S*_ and **Q**_*P*_ are the variables in the systemic and pulmonary circulatory systems, respectively. We provide the details and the parameters applied in this study in Supplementary Material 2.2.

The time integration of the combined system composed of Eqs 23–28 were performed with the Newmark-beta scheme:

$$\{\begin{array}{ll} \;{\dot{U}}_{T+\Delta T}={\dot{U}}_{T}+\Delta T(\gamma^{\ddot{U}}T+\Delta T+(1-\gamma ){\ddot{U}}_{T}) & \left(30\right)\\ U_{T+\Delta T}=U_{T}+\Delta T{\dot{U}}_{T}+\Delta T^{2}(\text{β}{\ddot{U}}_{T+\Delta T}+(1/2-\text{β}){\ddot{U}}_{T}) & \left(31\right)\\ \;R(U_{T+\Delta T},\;{\dot{U}}_{T+\Delta T},{\ddot{U}}_{T+\Delta T})=0. & \left(32\right) \end{array}$$

where the vector ***U*** contains all variables as follows:

(33) $$\text{U}=\left(\begin{array}{c} \begin{array}{c} \text{u}\\ p\\ P_{L} \end{array}\\ P_{R}\\ {}*\\ {}*\\ {}*\\ {}*\\ {}*\\ {}* \end{array}\right),\dot{\text{U}}=\left(\begin{array}{c} \begin{array}{c} \dot{\text{u}}\\ {}*\\ {}* \end{array}\\ {}*\\ F_{M\,I}\\ F_{A\,O}\\ F_{P\,A}\\ F_{T\,R}\\ {\text{Q}}_{P}\\ {\text{Q}}_{S} \end{array}\right),\ddot{\text{U}}=\left(\begin{array}{c} \begin{array}{c} \ddot{\text{u}}\\ {}*\\ {}* \end{array}\\ {}*\\ {}*\\ {}*\\ {}*\\ {}*\\ {\dot{\text{Q}}}_{P}\\ {\dot{\text{Q}}}_{S} \end{array}\right),$$

and the function *R* involves all the equilibrium and constraint conditions in Eqs 23–28. The missing components denoted by “^∗^” in Eq. 33 do not appear in the function ***R***. Although these components are calculated following the rules of time interpolation in Eqs 30, 31, they do not have any physical meaning.

Eqs 30–32 are simultaneously solved implicitly using Newton iterations as follows:

Set the initial guess: ${\text{U}}_{T+\Delta \,T}^{\left(0\right)}={\text{U}}_{T}$,${\dot{\text{U}}}_{T+\Delta \,T}^{\left(0\right)}={\dot{\text{U}}}_{T}$, ${\ddot{\text{U}}}_{T+\Delta \,T}^{\left(0\right)}={\ddot{\text{U}}}_{T}$

Iterate *k* = 0,1,2,⋯ until ||**R**^(*k*)^||≤ε

Compute the residual and the stiffness matrix:

$${\text{R}}^{\left(k\right)}=\text{R}\,\left({\text{U}}_{T+\Delta \,T}^{\left(k\right)},{\dot{\text{U}}}_{T+\Delta \,T}^{\left(k\right)},{\ddot{\text{U}}}_{T+\Delta \,T}^{\left(k\right)}\right)$$

$${\text{K}}^{\left(k\right)}=\frac{\partial \text{R}}{\partial \text{U}},{\text{C}}^{\left(k\right)}=\frac{\partial \text{R}}{\partial \dot{\text{U}}},{\text{M}}^{\left(k\right)}=\frac{\partial \text{R}}{\partial \ddot{\text{U}}}$$

Solve the linear system:

$$\left({\text{M}}^{\left(k\right)}+\gamma \,\Delta \,T\,{\text{C}}^{\left(k\right)}+\beta \,\Delta \,T^{2}\,{\text{K}}^{\left(k\right)}\right)\,\Delta \,{\ddot{\text{U}}}^{\left(k\right)}=$$

$$\left\{ \begin{array}{c} -{\text{R}}^{\left(0\right)}-{\text{C}}^{\left(0\right)}\,\Delta \,T\,{\ddot{\text{U}}}_{T}-{\text{K}}^{\left(0\right)}\,\left(\Delta \,T\,{\dot{\text{U}}}_{T}+\frac{1}{2}\,\Delta \,T^{2}\,{\ddot{\text{U}}}_{T}\right),k=0\\ -{\text{R}}^{\left(k\right)},k\geq 1 \end{array}\right.$$

Update:

$${\ddot{\text{U}}}_{T+\Delta \,T}^{\left(k+1\right)}={\ddot{\text{U}}}_{T+\Delta \,T}^{\left(k\right)}+\Delta \,{\ddot{\text{U}}}^{\left(k\right)}$$

$${\dot{\text{U}}}_{T+\Delta \,T}^{\left(k+1\right)}=\left\{ \begin{array}{c} {\dot{\text{U}}}_{T+\Delta \,T}^{\left(0\right)}+\Delta \,T\,\left(\gamma \,\Delta \,{\ddot{\text{U}}}^{\left(k\right)}+{\ddot{\text{U}}}_{T}\right),k=0\\ {\dot{\text{U}}}_{T+\Delta \,T}^{\left(k\right)}+\gamma \,\Delta \,T\,\Delta \,{\ddot{\text{U}}}^{\left(k\right)},k\geq 1 \end{array}\right.$$

$${\text{U}}_{T+\Delta \,T}^{\left(k+1\right)}\,\left\{ \begin{array}{c} {\text{U}}_{T+\Delta \,T}^{\left(0\right)}\,\Delta \,T\,{\dot{\text{U}}}_{T}+\Delta \,T^{2}\,\left(\beta \,\Delta \,{\ddot{\text{U}}}^{\left(0\right)}+1/2\,{\ddot{\text{U}}}_{T}\right),k=0\\ {\text{U}}_{T+\Delta \,T}^{\left(k\right)}+\Delta \,T^{2}\,\beta \,\Delta \,{\ddot{\text{U}}}^{\left(k\right)},k\geq 1 \end{array}\right.$$

Because the initial guesses ${\text{U}}_{T+\Delta \,T}^{\left(0\right)}$, ${\dot{\text{U}}}_{T+\Delta \,T}^{\left(0\right)}$, and ${\ddot{\text{U}}}_{T+\Delta \,T}^{\left(0\right)}$ do not fulfill the interpolation rules in Eqs 30, 31, whereas the solutions after that do, different right-hand sides and update rules are applied. The key issue in the Newton iteration is the treatment of active stress **S**_*act*_ in the computation of stiffness matrix **K** = ∂⁡**R**/∂⁡**U**. The active force vector and the stiffness matrix associated with the active stress tensor **S**_*act*_ on an element *e* are given by

(34) $${\text{F}}_{a\,c\,t,e}=\int_{e}T_{a\,c\,t}\,\frac{\partial \lambda }{\partial {\text{u}}_{e}}\,d\Omega$$

**FIGURE 4:**
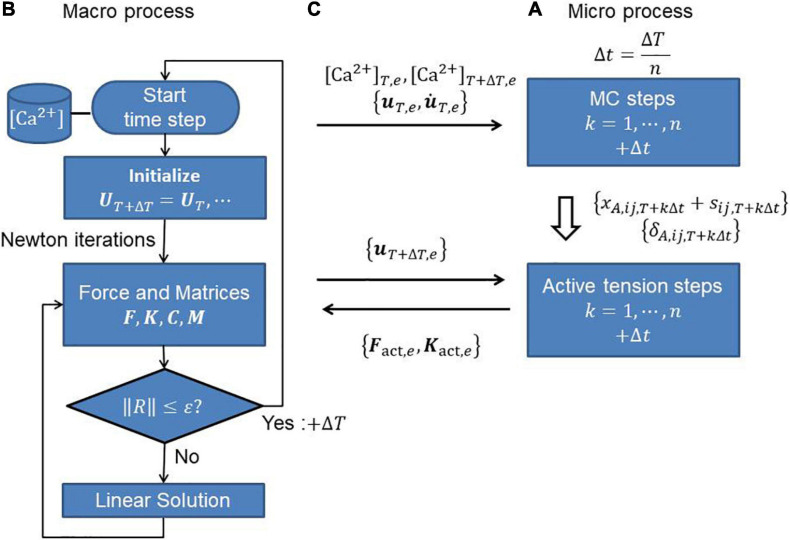
**(A)** Micro and **(B)** macro processes, and **(C)** communications between them to perform the MusAsi scheme. Before the Newton iterations, the displacement **u**_*T*,*e*_ and its time derivative ${\dot{\text{u}}}_{T,e}$ on individual elements {*e*} are sent from the macro process to the micro process to perform the MC steps in the micro process. In the Newton iterations, the current displacement **u**_*T* + Δ*T*,*e*_ is sent to the micro process to compute the active force vector **F**_act,*e*_ and the active stiffness matrix **K**_act,*e*_ on each element *e*.

(35) Ka⁢c⁢t,e=∫e∂⁡Ta⁢c⁢t∂⁡λ⁢(∂⁡λ∂⁡ue)T⁢(∂⁡λ∂⁡ue)⁢dΩ+∫eTa⁢c⁢t⁢∂2⁡λ∂⁡ue2⁢dΩ

where the derivatives of stretch λ with respect to nodal displacements **u**_*e*_ of the element *e* are given by Eqs 19, 22. In the MusAsi scheme, the active tension *T*_*act*_ and its derivative ∂⁡*T*_*act*_/∂⁡λ must be computed from the current value of λ_*T* + Δ*T*_ as defined by Eqs 8, 12, respectively, in every Newton iteration step. The processing flows and the data transfers between the macro and micro processes are shown in Figure 4.

### Computer Resource and the FE Model

The tested biventricular FE model consisted of 45,000 tetrahedral elements, where the MINI(5/4c) element (Brezzi and Fortin, 1991) was adopted to avoid instability caused by the nearly incompressible condition in Eq. 24. Although the higher-order interpolation of MINI elements was applied to the displacement *u* to evaluate the integration associated with the passive stress tensor, standard linear interpolation ignoring the central node was adopted for active stress. Thus, it was sufficient to assign one half-sarcomere model to each element. The fiber-sheet architecture was constructed by applying the optimization algorithm in our previous work (Washio et al., 2020). The computations were performed using 20 nodes (320 cores) of a parallel computer system (Intel Xeon E-2670 [2.6 GHz], 16 cores/node; Intel, Santa Clara, CA, United States). In the typical MusAsi scheme in which 16 filaments (*N_F_* = 16) were assigned to each half-sarcomere model, the computational time was about 1.26 h per heartbeat with Δ*T* = 1.25ms, and Δ*t* = 5μs. The MS steps and active tension integration steps (Figure 4A) in the micro process took 0.58 and 0.42 h, respectively. The remaining computational time was almost occupied with the linear solutions (Washio and Hisada, 2011; Kariya et al., 2020) in the macro process (Figure 4B). Because 38 myosin molecules were arranged in each filament, 27 million myosin molecules were used in total.

The heart rate was set to 60 beats per minute, and the Ca^2+^-transient generated by the midmyocardial cell model proposed by ten Tusscher and Panfilov (2006) was applied (Figure 2C). Transmural delays were used that were determined by the distances from the endocardial surfaces of the left and right ventricles under a transmural condition velocity of 52 cm/s, as measured by Taggart et al. (2000).

From the numerical results, the output work from the aortic valve was evaluated as

(36) $$W_{o\,u\,t}=\int_{0}^{T_{C}}F_{A\,O}\cdot P_{L}\,dT,$$

where *T*_*C*_ = 1 s is the cardiac cycle period. ATP consumption was also calculated by counting the transients to N_*XB*_ from XB_*PostR2*_ or N_*ATP*_ (Figure 2A).

## Results

### Accuracy in Overall Cardiac Outcomes

**FIGURE 5:**
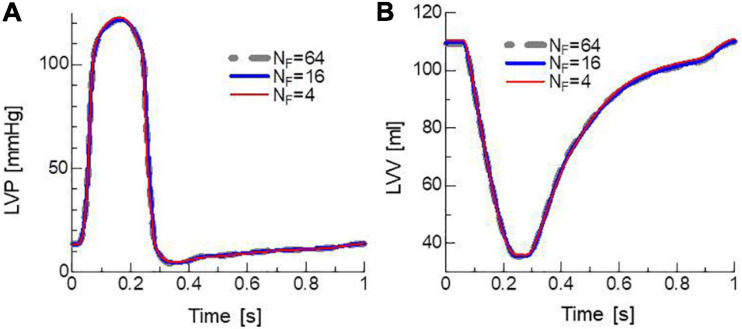
Time transients of **(A)** left ventricular pressure (LVP) and **(B)** volume (LVV) for *N*_*F*_ = 4 (thin red lines), 16 (medium thick blue lines), and 64 (thick gray broken lines). Δ*T* = 1.25 ms and Δ*t* = 5 μs were applied. These almost equal time transients were taken from the third cycle after the initial process of blood filling.

The influence of the number of filaments *N_F_* imbedded in one element on cardiac outcomes, such as pressure and volume, are shown in Table 1A and Figure 5. In the simulations, Δ*T* = 1.25 ms and Δ*t* = 5 μs were applied so that 250 MC steps were performed within a single FE step. Although there was a little difference in the overall time transients of pressure and volume, even for *N_F_* = 4, the difference between the minimum and maximum of these variables from *N*_*F*_ = 64 was less than 1%. The difference in ATP consumption from *N*_*F*_ = 64 was slightly larger than that of pressure and volume. However, it was also less than 1% for *N_F_* = 16. Thus, it seemed to be sufficient to use 16 filaments to obtain the overall cardiac outcomes.

**TABLE 1:** Influence of the filament number *N*_*F*_ **(A)** and FE time step size Δ*T* **(B)** on overall pumping performance and computational time.

(A) Influence of *N*_*F*_					
*N*_*F*_	SV/EDV [ml]	EDP/P_*max*_ [mmHg]	W_*out*_/ATP [J]	dP/dT_*max*_ [mmHg/s]	Time [h]
4	74.4/110.9	13.7/122.8	1.17/5.09	4291	0.75
8	74.0/110.5	13.6/122.1	1.16/4.99	4237	0.94
16	74.0/110.1	13.7/121.9	1.15/4.94	4341	1.26
32	74.0/110.0	13.7/122.0	1.15/4.92	4295	1.88
64	73.8/110.0	13.7/121.9	1.15/4.90	4275	3.19

**(B) Influence of Δ*T***					

Δ*T*	SV/EDV [ml]	EDP/P_*max*_ [mmHg]	W_*out*_/ATP [J]	dP/dT_*max*_ [mmHg/s]	Time [h]

Δ*T*_0_	74.0/110.1	13.7/121.9	1.15/4.94	4341	1.26
Δ*T*_0_/2	73.6/110.2	13.7/121.5	1.14/4.96	4287	1.80
Δ*T*_0_/4	73.6/110.2	13.7/121.4	1.14/4.95	4267	2.92

*SV: stroke volume, EDV: end-diastolic volume, EDP: end-diastolic pressure, P_*max*_: maximal pressure, W_*out*_: work at output, ATP: ATP consumption, time: computational time for one cycle. These values were taken from the third cycle after the initial process of blood filling. ATP consumption in the septum was included. Δ*T* = 1.25 ms and Δ*t* = 5 μs were applied in (A). *N*_*F*_ = 16 and Δ*T*_0_ = 1.25 ms were applied in (B).*

The influence of the FE time step size Δ*T* on the overall cardiac outcomes was also examined, as shown in Table 1B. The baseline of the MC time step size was given by Δ*t*_0_ = 5 μs, and the number of MC steps *n* performed within a single FE step was determined by

(37) $$n\equiv \left\lfloor \frac{\Delta \,T-0.5\,\Delta \,t_{0}}{\Delta \,t_{0}}\right\rfloor +1,$$

where “⌊⌋” represents the floor function that rounds down after the decimal point. Therefore 250, 125, and 63 MC steps were performed with Δ*T* = Δ*T*_0_, Δ*T*_0_/2, and Δ*T*_0_/4 (Δ*T*_0_ = 1.25 ms), respectively. As with the number of filaments *N_F_*, the difference with Δ*T* was sufficiently small. As the computational time increased, Δ*T* decreased because the total Newton iterations and the communication between the MC and FE models increased. This result suggests that the choice Δ*T* = 1.25 ms was sufficiently good and preferable in terms of the computational cost.

**FIGURE 6:**
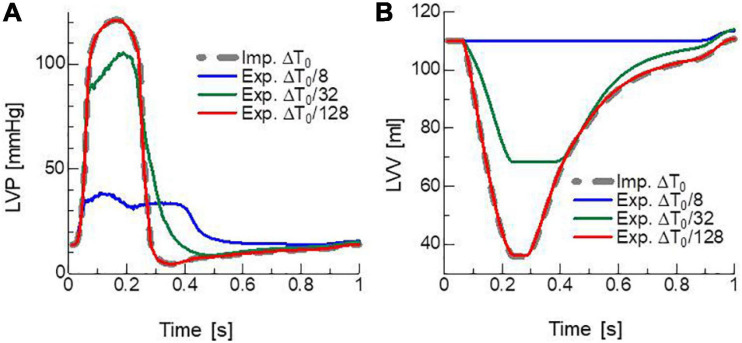
Time transients of **(A)** left ventricular pressure (LVP) and **(B)** volume (LVV) obtained by the MusAsi with the FE time step size Δ*T* = Δ*T*_0_ (thick gray broken lines) and the explicit active tension approach with the finer time steps Δ*T* = Δ*T*_0_/8 (blue lines), Δ*T*_0_/32 (green lines), and Δ*T*_0_/128 (red lines).

To confirm the necessity of the implicit approach for active tension, an explicit approach with various sizes of the FE time step Δ*T* was tested (Figure 6). In the explicit approach, only the calculation of active tension was modified so that it was determined using the strains $\left\{{\tilde{x}}_{i\,j,T+k\,\Delta \,t}\right\}$ in Eq. 7 calculated in the MC steps from the stretch λ and stretch rate $\dot{\lambda }$ at time *T* instead of {*x*_*ij*,*T* + *k*Δ*t*_} in Eq. 9 calculated from the stretch λ at time *T* + Δ*T*. In this explicit case, only the second term on the right-hand side in Eqs 21, 35 was used to construct the stiffness matrix because neither the stretch λ or stretch rate $\dot{\lambda }$ at time *T* + Δ*T* was involved in the definition of *T*_*act*_ at time *T* + Δ*T*. Although there was no breakdown of the Newton iterations with the explicit approach, incorrect results appeared at certain times during the contraction phase. The smaller the time step size Δ*T*, the later the time at which the wrong result appeared. Additionally, finally, almost the same result as the implicit approach with Δ*T* = Δ*T*_0_ = 1.25 ms was reproduced with quite a small time step Δ*T* = Δ*T*_0_/128∼10 μ**s**. This result supports both the numerical accuracy and computational efficiency of the MusAsi scheme because the stable explicit approach with Δ*T* = Δ*T*_0_/128 required communication between the micro and macro processes and the linear solution in the macro process every MC step (*n* = 2) and, thus, a single beat computation took 70 h, whereas the MusAsi took only 1.26 h with *n* = 250 without loss of accuracy.

### Accuracy of Local Dynamics

**FIGURE 7:**
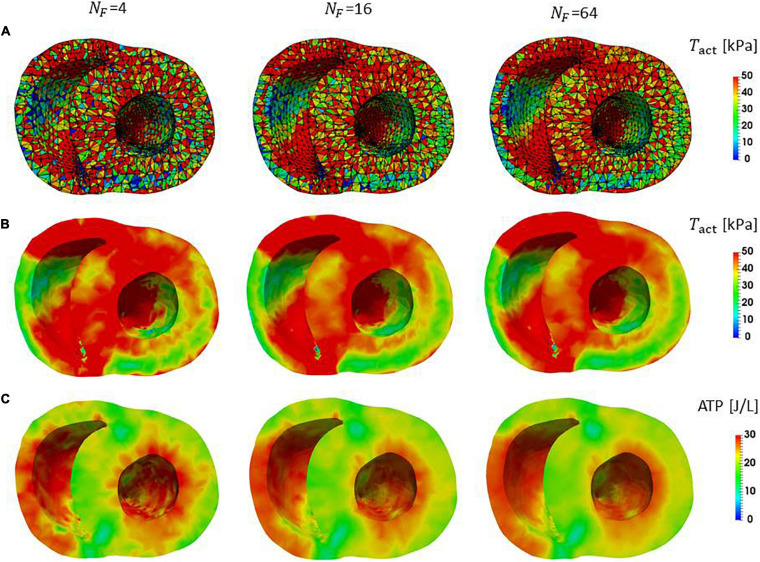
Distributions of active tension and ATP consumption at *T* = 0.2 s in the contraction phase in the middle cross-section perpendicular to the long axis. **(A)** The active tensions calculated in the individual elements are shown. The lines indicate the segmentation to elements. The black lines are element boundaries. **(B)** The active tensions in elements were averaged on the nodes in the FE mesh. **(C)** The ATP consumption in the interval [0.0 s, 0.2 s] for the individual elements was averaged on the nodes in the FE mesh.

**FIGURE 8:**
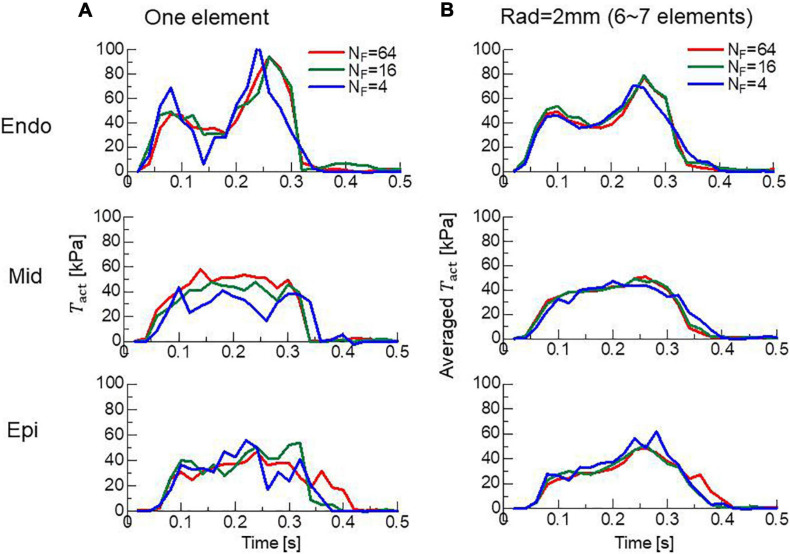
Time transients of local active tensions at the Endo, Mid, and Epi sites in the lateral left ventricular wall. **(A)** The active tensions *T*_*act*_ at a single element are plotted for *N*_*F*_ = 4 (blue lines), 16 (green lines), and 64 (red lines). **(B)** The averaged values with surrounding elements within 2 mm are plotted.

In clinical applications, not only the overall cardiac outputs, but also the local mechanical load and energy consumption are important for predicting a remote prognosis. To confirm the accuracy of local dynamics, the influence of the filament number *N_F_* on the distribution of active tension *T*_*act*_ and ATP consumption at the peak of the systolic phase were examined (Figure 7). Although the discontinuities of active tensions at element boundaries was slightly noticeable, even for *N_F_* = 64 (Figure 7A), it became inconspicuous when the elementwise variables were averaged at the nodes for *N*_*F*_≥16 (Figures 7B,C). The distributions of the active tension values and the ATP consumption values over the entire cycle are shown in Supplementary Video 1. A more detailed comparison of the time transients of active tensions at a single element further indicated that the choice *N_F_* = 16 was sufficient for analyzing the local mechanical load (Figure 8).

It is somewhat counter-intuitive that the highest ATP consumption is not happening in the regions of highest active tension production (Figures 7B,C). Since the ATP consumption in Figure 7C is the cumulative value over the time interval [0.0 s, 0.2 s], it is difficult to find temporal relationship with the active tension. In Supplementary Material 3, the active tension, the stretch rate, and the ATP consumption rate at *T* = 0.2 s are shown. Here, due to the force-velocity relationship (Figure 10B), the higher the shortening velocity (negative stretch rate) is, the smaller the active tension gets. Because the shortening of half-sarcomere shifts the rod strain distribution to the negative direction (Figure 1C) resulting in the facilitation of power stroke (*h*_*f,1*_ and *h*_*f,2*_ in Figure 13A), the higher the shortening velocity is, the higher the ATP consumption rate gets. Therefore, the lowest ATP consumption rate is happening in the region of highest active tension production when the stretch is relatively uniform over the region.

### Sensitivity of the Nearest Neighbor Co-operative Parameter

**FIGURE 9:**
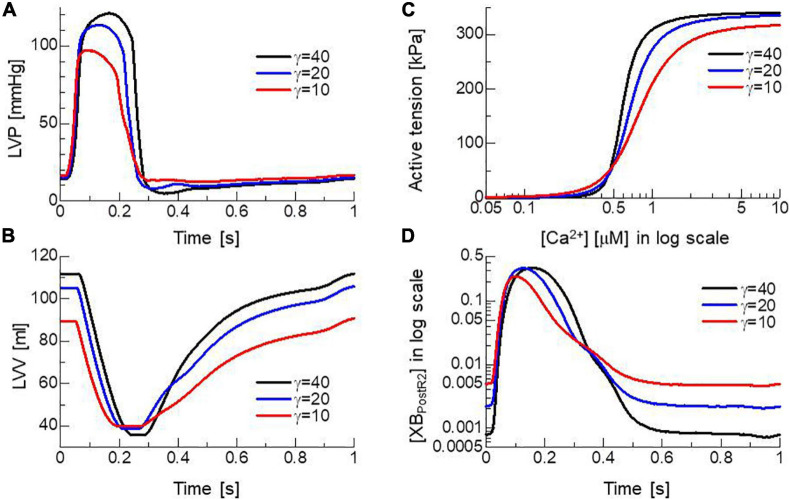
Effects of the nearest neighbor co-operative parameter γ on the time transients of **(A)** left ventricular pressure (LVP), **(B)** volume (LVV), **(C)** the force-pCa relationship under the unloaded sarcomere length, and **(D)** time transient of state ratio [XB_*PostR2*_] in the logarithmic scale. The black, blue, and red lines indicate γ = 40, 20, and 10, respectively.

To confirm the importance of the neighboring co-operative mechanism for the relaxation phase in the cardiac cycle, pumping performances were compared for different co-operative parameters γ=40, 20, and 10 (Figures 9A,B). Because the Ca^2+^-concentration did not disappear, even in the diastolic phase (Figure 2C), active tensions were not completely removed with the impaired co-operativity (Figure 9C). Thus, the insufficient drop of left ventricular pressure (LVP) blocked the filling of blood through the mitral valve. The time transients of [XB_*PostR2*_] indicate that even a small binding population less than 1% was likely to hamper the extension of the ventricular cavity (Figure 9D).

### Sensitivity of the Power and Reverse Stroke Rate Constants

To examine the capability of MusAsi to reflect the stochastic behavior of the power and reverse strokes on cardiac outcomes, we examined the following alternative of the load dependent power stroke model, which we adopted in our previous work to reproduce the SPOC of a single myofibril of rabbit iliopsoas muscle (Washio et al., 2019):

$$\left\{ \begin{array}{c} h_{f,i}\,\left(x\right)=g_{i}\,\text{exp}\,\left(\frac{E_{i-1}+W\,\left(x\right)-E_{i}-W\,\left(x+s_{i}/2\right)}{k_{B}\,T}\right)\;\left(38\right)\\ h_{b,i}\left(x+s_{i}\right)=g_{i}\text{exp}\left(\frac{W\,\left(x+s_{i}\right)-W\,\left(x+s_{i}/2\right)}{k_{B}\,T}\right).\;\left(39\right) \end{array}\right.$$

This model originated from the Kramers escape theory (Kramers, 1940; Scherer and Fischer, 2017), in which the rate constants were defined by the Boltzmann factor associated with the height of the energy barrier from the origin, whereas the previous definition in Eqs 2, 3 used the strain energy at the destination. In Eqs 38, 39, *W*(*x* + *s*_*i*_/2) was introduced to represent the contribution of the strain energy at the energy barrier that was assumed to be located at the mid strain (Washio et al., 2017). The contribution of the free energy of the myosin head at the barrier was included in the constant *g_i_*. In this study, *g*_1_ = 20 [1/s] and *g*_2_ = 0.1 [1/s] were adopted, as in our previous work (Washio et al., 2019). Furthermore, the rate constant (*k*_*np*_ in Figure 2A) from the non-binding state N_*XB*_ to the weak binding state P_*XB*_ was multiplied by the factor 1.1 so that the same maximal pressure (P_*max*_: the maximum of LVP) was achieved by both models. Hereafter, we call the power stroke models of Eqs 2, 3 and of Eqs 38, 39) the destination strain energy (DSE) model and barrier strain energy (BSE) model, respectively.

**FIGURE 10:**
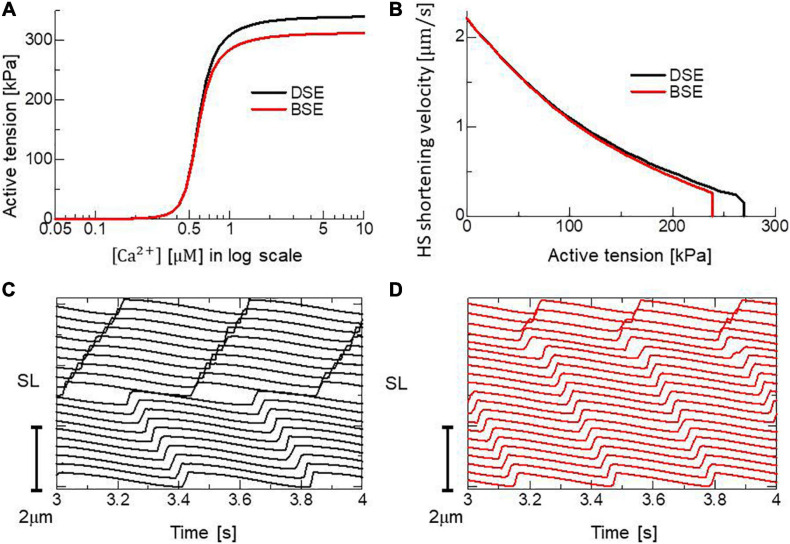
Comparisons of the DSE and BSE models in **(A)** the force-pCa relationship under the unloaded sarcomere length, **(B)** the force-velocity relationship at [Ca^2+^] = 0.7 μM. Both the **(C)** DSE and **(D)** BSE models reproduced the SPOC for 20 sarcomeres in a single myofibril under the constant Ca^2+^ concentration ([Ca^2+^] = 0.3 μM).

**FIGURE 11:**
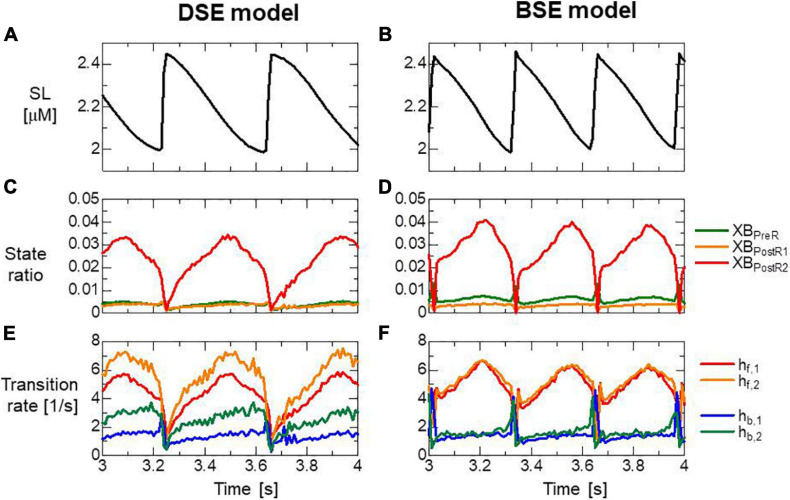
**(A,B)** Time transients of the sarcomere length (SL), **(C,D)** ratio of binding states, and **(E,F)** transition rates between the binding states for the half-sarcomere model imbedded at the center of the myofibril model consisting of 20 sarcomeres during the SPOC with [Ca^2+^] = 0.3 μM. Panels **(A,C,E)** and panels **(B,D,F)** are the results of the DSE and BSE models, respectively. **(E,F)** Transition rates were calculated by dividing the total number of transitions per unit time by the total number of myosin molecules.

**FIGURE 12:**
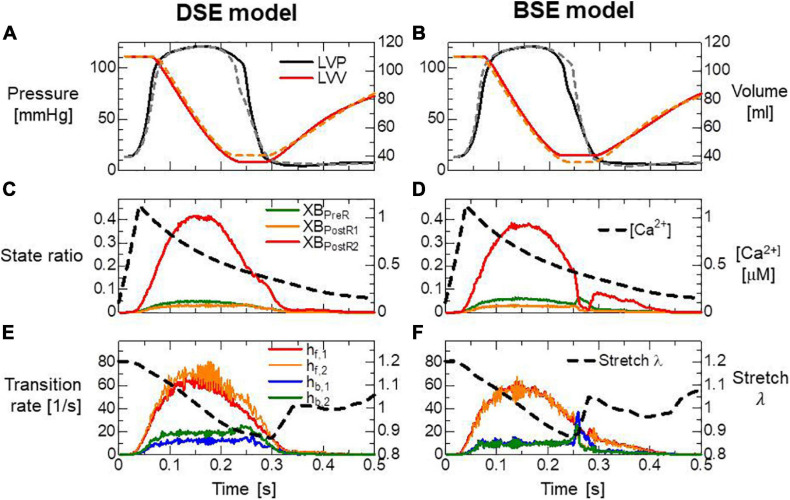
**(A,B)** Time transients of the left ventricular pressure (LVP) and volume (LVV) of the FE ventricle model, **(C,D)** the ratio of binding states to the Ca^2+^ concentration, and **(E,F)** the transition rates between the binding states and the stretch given by averaging the half-sarcomere models imbedded at the endo lateral left ventricular wall (elements inside a sphere of 3 mm radius). Panels **(A,C,E)** and panels **(B,D,F)** are the results of the DSE and BSE models, respectively. In panels **(A,B)**, the broken lines indicate the results of counterparts. The transition rates in panels **(E,F)** were calculated by dividing the total number of transitions per unit time by the total number of myosin molecules.

Both models reproduced similar tendencies in the force-pCa relationship (Figure 10A) and the force-velocity relationship (Figure 10B) though the active tensions of the BSE model were slightly smaller than that of the DSE model for a large Ca^2+^ concentration (>0.5 μM) or a small half-sarcomere shorting velocity (<1 μm/s). The SPOCs on the single myofibril model consisting of 40 half-sarcomeres under the constant [*Ca*^2 +^] = 0.3μM were also reproduced by both models (Figures 10C,D). However, their periods and amplitudes were different (Figures 11A–F). In particular, the remarkable increases of two reverse stroke rates, which we called the avalanche of reverse strokes in our previous works (Washio et al., 2017, 2019), were observed for the two reverse rates at the lengthening in the BSE model, whereas such an increase was slightly recognized only in the reverse stroke rate from XB_*P*__*ostR1*_ and XB_*P*__*reR*_ in the DSE model. Furthermore, the ratios of the reverse rates to the forward rates were higher for the DSE model than for the BSE model. These differences in the SPOCs of the two models were also recognized in the numerical results for the ventricle FE model (Figures 12A–F). The local contraction duration of the DSE model was longer than that of the BSE model (Figures 12C,D) as the difference in the SPOC periods between the two models (Figures 11A,B). The rise and drop of LVP for the BSE model were slower than for the DSE model. In particular, for the BSE model, there was a small rebound of the binding population once after the binding myosin molecules almost disappeared. Although the rebound population was small, the binding myosin molecules clearly hampered the drop of LVP. In the BSE model, though the quick lengthening of sarcomere accompanying the avalanche of reverse strokes was observed, it was not reflected in the rapid transition from the systolic phase to the diastolic phase because of the rebounds.

**FIGURE 13:**
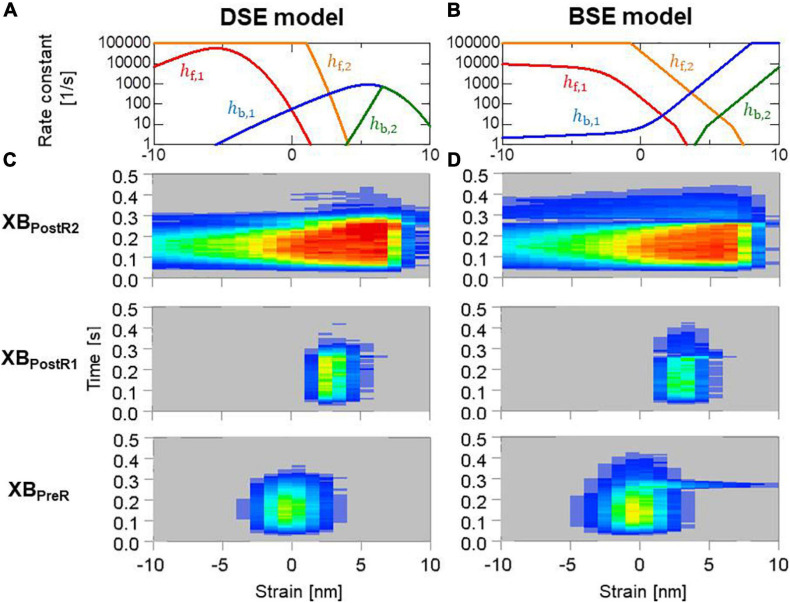
**(A,B)** Rate constants in the logarithmic scale between the binding states, and **(C,D)** time transients of the distribution of binding states in the rod strain space in the half-sarcomere models imbedded at the endo lateral left ventricular wall (elements inside a sphere of 3 mm radius). Red and blue indicate high and low density, respectively. Panels **(A,C)** and panels **(B,D)** are for the DSE and BSE models, respectively.

In Figure 13, the distribution of binding states on the rod strain space *x* ∈ [−10nm,10nm] on the elements at the endo lateral left ventricular wall are provided with the rate constants. The large magnitude of the reverse stroke rates (*h*_*b*,1_,*h*_*b*,2_) of the BSE model (Figure 13B) caused an avalanche of reverse strokes and local quick lengthening (Figure 12F). The small distribution of the rebound after lengthening was recognized (Figure 13D). Note that the MusAsi scheme allowed us to directly couple such distributions of rod strains with wall motion in the biventricular FE model.

## Discussion

### Practicality in Clinical Applications

Using 320 cores of a conventional parallel computer system, one cardiac cycle of the MusAsi scheme could be computed within 1.5 h for a sufficiently fine FE biventricle model consisting of 45,000 tetrahedral elements. Accuracy and robustness were also confirmed through sensitivity tests with the various parameters of sample numbers and time step sizes. In fact, we have already applied the approach to follow-up verifications of practical clinical problems (Kariya et al., 2020; Masuda et al., 2021). In these cases, pumping performance after operations was predicted not only using the standard indices, such as LVP, ejection fraction (EF), and stroke volume (SV), but also the energy consumption. Now, we are moving to the next stage of applying our simulator using the MusAsi scheme in prospective clinical trials in an ongoing project on congenital heart disease.

### Relaxation Mechanism

How the rapid drop of LVP can be achieved for the intracellular Ca^2+^ transient with a slow attenuation (Figure 2C) may be still a controversial issue. Furthermore, the population of binding myosin molecules must almost vanish in the relaxation phase, whereas nearly 10% of the maximum is left in the Ca^2+^ concentration (Figures 12C,D). In our model, the latter problem was resolved by adopting the nearest neighbor co-operative mechanism in transitions between the non-binding state and weak binding state (Figures 2A, 9), whereas the former problem was resolved by the reverse stroke mechanism introduced by the load-dependent power stroke model (Figure 12). The MusAsi scheme enabled the stochastic cross-bridge mechanisms and the macroscopic dynamics to be directly coupled, and we confirmed that these molecular mechanisms work efficiently to achieve the physiological relaxation of cardiac muscle in the beating cycle. A comparison of relaxation in the two power stroke models indicated the usefulness of the MusAsi scheme as a basic research tool in fields that study the role of molecular-level observation in the heartbeat (Figures 10–13). In our previous work (Washio et al., 2018) in which we directly coupled the Langevin dynamics model and the FE ventricle model, we detected the same problem of the slowed LVP drop caused by the rebounds observed in the BSE model. This problem was resolved by introducing the trapping mechanism that inhibits the reverse strokes when the rod strains increased quickly over a certain threshold. The trapping mechanism may have similar effects to the DSE model in which the reverse rates are drastically reduced for large strains (Figure 13A). Furthermore, the experimental measurements made by Hwang et al. (2021) revealed a higher frequency of backward steps at lower loads of the cardiac myofilaments than those of fast skeletal myofilaments like the higher reverse rate *h*_*b,1*_ of the DSE model than that of the BSE model (Figures 13A,B). Our numerical results suggest that such a characteristic of the reverse rate brings the benefit to the cardiac myofilaments for quick relaxation. Note that achieving the quick relaxation of muscle is crucial in simulations of congenital heart disease because heart rates are more than a hundred, in most cases.

### Significance of Active Stiffness

A key factor for stability in the MusAsi scheme is the implicit treatment of the active stress tensor **S**_*act*_ in the standard FE framework using Newton iterations. Assuming a constant stiffness *k*_*rod*_ per binding myosin molecule and a binding ratio *R_B_* in the half-sarcomere model, the macroscopic axial stiffness coefficient *K_A_* in the fiber orientation for the active tension in Eq. 8 is estimated as

(40) $$K_{A}=\frac{R_{S}}{S\,A_{0}}\,R_{B}\,2\,N_{M}\,k_{r\,o\,d}\,\frac{S\,L_{0}}{2}=104.2\,R_{B}\,\text{MPa}$$

with the adopted parameters: sarcomere volume ratio *R*_*S*_ = 0.5, cross-sectional area *SA*_0_ = 693nm^2^ (Sato et al., 2013), number of accessible myosin molecules 2*N*_*M*_ = 76 per thin filament, spring coefficient of the myosin rod with positive strains *k*_*rod*_ = 2*pN*/*nm*, and HSL *SL*_0_/2=0.95μm. Because we applied the viscosity coefficient μ_*S*_ = 36.66*Pa*⋅*s*, the stability condition for the time step size in the explicit approach is roughly estimated as

(41) $$\Delta \,T\leq \frac{\mu_{S}}{K_{A}}∼\frac{0.35}{R_{B}}\,\mu \,s.$$

Note that the stiffness coefficients for passive stress are in the order of kPa for strains less than 0.2 (see Supplementary Material 2.1 for details of the passive material parameters). Therefore, the contribution of passive stiffness, which is dealt with implicitly, is negligible compared with active stiffness in Eq. 40, even if only a few percent of myosin molecules are in binding states (*R*_*B*_∼0.02). The above estimations of the limitation of the time step size in the explicit approach for active tension are good fit for the instability depending on the time step size in Figure 6. The stiffness estimation in Eq. 40 also justifies the significant influence of a single binding myosin molecule contained in the half-sarcomere model (*R_B_*∼1/38) regarding hampering the diastole as observed in Figure 9.

### Future Directions

In the one-dimensional (1D) half-sarcomere model adopted in this study, the characteristics of the realistic 3D regular arrangements of myosin molecules on the thick filament and the binding sites on the double spirals on the thin filament (Hussan et al., 2006) were not taken into account. These geometrical parameters are likely to have been optimized in the process of evolution. Thus, they may have a significant influence on the rate constant of binding transitions from the P_*XB*_ state to the XB_*PreR*_ state and the initial rod strains that were provided probabilistically from the Boltzmann distribution exp(−*W*(*x*)/*k*_*B*_*T*) of strain energy *W* in this study. The modeling of active stress was also limited only to the fiber orientations in this study. However, actin filaments are pulled not only in the longitudinal direction of the sarcomere but also in the lateral direction by myosin rods. These limitations should be removed by extending the MusAsi scheme from the 1D model to an appropriate 3D model in our future work.

In this study, we applied the MusAsi scheme to the simplified model to focus on the impact of the properties of contractile proteins on the macroscopic outcomes. In the heart model of our previous work (Kariya et al., 2020) in which a similar approach for coupling MC and FEM simulations (Washio et al., 2016) has been used, three species of ventricular myocytes, i.e., endocardial, mid-myocardial, and epicardial cells, were implemented. Therefore, we believe that the current scheme will also work in a model implemented with the realistic electrophysiology. In that work, we also assumed that the heart walls were surrounded by the pericardium, which was fixed in space by the planar springs, and we incorporated the impact of pericardial pressure that was generated based on volume conservation of pericardial liquid. It is expected that the negative pericardial pressure also facilitates the drop of LVP at the early diastole. The contributions of these more realistic boundary conditions will be evaluated in future studies that should also take the pre-stress of the myocardium into account.

## Data Availability Statement

The original contributions presented in the study are included in the article/Supplementary Material, further inquiries can be directed to the corresponding author/s.

## Author Contributions

TH, SS, and MW designed the project. J-IO and KY prepared the input data for the computer simulations. TW and KY developed the multiple time step active stiffness scheme, ran the simulations, analyzed the simulation data, and wrote the manuscript with input from SS, TH, and MW. TW, J-IO, and KY developed the simulation code with input from SS, TH, and MW. All authors contributed to the article and approved the submitted version.

## Conflict of Interest

TW, J-IO, SS, and TH were employed by UT-Heart Inc. KY and MW were employed by Fujitsu Japan Ltd.

## Publisher’s Note

All claims expressed in this article are solely those of the authors and do not necessarily represent those of their affiliated organizations, or those of the publisher, the editors and the reviewers. Any product that may be evaluated in this article, or claim that may be made by its manufacturer, is not guaranteed or endorsed by the publisher.
