# Supplementary material for: A Multiple Step Active Stiffness Integration Scheme to Couple a Stochastic Cross-Bridge Model and Continuum Mechanics for Uses in Both Basic Research and Clinical Applications of Heart Simulation
Source: Front Physiol. 2021 Aug 13;12:712816. doi: 10.3389/fphys.2021.712816 (PMC8414591; doi:10.3389/fphys.2021.712816)
Supplement: Supplementary file 2 [file Data_Sheet_1.PDF]

Supplementary Material

A multiple step active stiffness integration scheme to couple a stochastic cross-bridge model and continuum mechanics for uses in both basic research and clinical applications of heart simulation

Kazunori Yoneda[1], Jun-ichi Okada[2,3], Masahiro Watanabe[1], Seiryo Sugiura[2], Toshiaki Hisada[2], Takumi Washio[2,3*]

[1]Section Solutions Division, Healthcare Solutions Development Unit, Fujitsu Japan Limited, 1-5-2 Higashishimbashi, Minato-ku, Tokyo, Japan

[2] UT-Heart Inc., Kashiwanoha Campus Satellite, 178-4-4 Wakashiba, Kashiwa, Chiba 277-0871, Japan

[3] Future Center Initiative, University of Tokyo, Kashiwanoha Campus Satellite, 178-4-4 Wakashiba, Kashiwa, Chiba 277-0871, Japan

*** Correspondence:**
Takumi Washio
washio@ut-heart.com

S1 MC model

S1.1 Control model of attachment and detachment

Here, the parameters used in this study are listed in Table S1.1. In our model, we assumed that the attachment, which represents the transition from the P_{XB} state to the XB_{PreR} state (Figure 2A), was allowed only in the single overlap region of the thin and thick filaments. We also assumed that the myosin molecules were arranged on a thick filament at regular intervals, except for the bare zone (B-zone). Therefore, the myosin head ($\#i$) was situated in the single overlapping region only if the following condition was fulfilled:

$$\max(LA - HSL, HSL - LA) \le \frac{LB}{2} + \frac{2(i - 0.5)}{n_M(LM - LB)} \le HSL \qquad \text{(S1.1)}$$

Here, the middle term is the distance from the centre of the sarcomere. LM, LB, and LA represent the lengths of the thick filament, the B-zone, and the thin filament, respectively. $HSL = \lambda \cdot SL_0/2$ is the half-SL for the stretching parameter λ. The parameters for the sarcomere geometry were determined from cardiac sarcomeres [1, 2, 3, 4].

The thin filament was divided into N_T segments, termed troponin/tropomyosin (T/T) units (Figure S1) Three states (Ca-off, Ca-on*, and Ca-on) were assumed by each T/T unit. The transitions between the states of the T/T unit were determined by the Ca^{2+} concentration $[Ca^{2+}]$ and the four

parameters $\overline{K}_{\text{on}}^{*}$, $\overline{K}_{\text{on}}$, $\overline{K}_{\text{off}}^{*}$, and $\overline{K}_{\text{off}}$, as shown in Figure 2B. Those four parameters were defined as follows:

$$\overline{K}_{\text{on}}^{*} = \begin{cases} K_{\text{on}}'^{*}, & \text{if there is an MH binding below,} \\ K_{\text{on}}^{*}, & \text{otherwise.} \end{cases}$$

$$\overline{K}_{\text{off}}^{*} = \begin{cases} K_{\text{off}}'^{*}, & \text{if there is an MH binding below,} \\ K_{\text{off}}^{*}, & \text{otherwise.} \end{cases}$$

$$\overline{K}_{\text{on}} = \begin{cases} K_{\text{on}}', & \text{if there is an MH binding below,} \\ K_{\text{on}}, & \text{otherwise.} \end{cases}$$

$$\overline{K}_{\text{off}} = \begin{cases} K_{\text{off}}', & \text{if there is an MH binding below,} \\ K_{\text{off}}, & \text{otherwise.} \end{cases}$$

Here, MH stands for the myosin head. The transitions between the N_{XB} and P_{XB} states (Figure 2A) were affected by the status of the T/T unit above it, via modifications of k_{np} and k_{pn}, as well as by the states of the neighbouring myosin heads through the integer ng. The value of ng $(= 0, 1, \text{or } 2)$ represents the number of neighbouring myosin heads in the P_{XB} state or the three bound states. The corresponding T/T unit index τ for the i-th myosin head is given by:

$$\tau = \left\lfloor \frac{0.5LB + (i - 0.5)S_M - (HSL - LA)}{S_T} \right\rfloor \quad \text{(S1.2)}$$

Here, "⌊ ⌋" stands for the floor function that rounds down after the decimal point. The parameter $S_M = 0.5(LM - LB)/N_M$ represents the spacing of the myosin heads, and $S_T = LA/N_T$ is the spacing of the T/T units. The corresponding T/T unit exists only if $1 \leq \tau \leq N_T$. Based on this correspondence, the factors k_{np} and k_{pn} of the rate constants were given by:

$$k_{\text{np}} = \begin{cases} \delta_{OV} K_{\text{np1}}, & \text{if the T/T unit above is in the Ca-on state,} \\ \delta_{OV} K_{\text{np0}}, & \text{otherwise.} \end{cases} \quad \text{(S1.3)}$$

$$k_{\text{pn}} = \begin{cases} K_{\text{pn1}}, & \text{if the T/T unit above is in the Ca-on state,} \\ K_{\text{pn0}}, & \text{otherwise.} \end{cases} \quad \text{(S1.4)}$$

Here, $\delta_{OV} = 1$ if the myosin head was located at the single overlapping region with the thin filament, otherwise $\delta_{OV} = 0$. The factors γ^{ng} and γ^{-ng} $(\gamma = 40)$ represent the nearest-neighbour cooperativity of the myosin heads, as reported by Rice [3], which plays an important role for the force-pCa relationship. We assumed that one thin filament in the three-dimensional arrangement corresponds to two thin filaments in our half-sarcomere model. This is because we assumed that cooperative behaviour exists along the tropomyosin molecules wrapped around the thin filament in a double spiral fashion, and one spiral was modelled in our half-sarcomere. The constants K_{np0}, K_{np1}, K_{pn0}, and K_{pn1} were determined from Q, K_{basic}, and μ, as follows:

$$K_{\text{np0}} = F_K(SL)\frac{QK_{\text{basic}}}{\mu},\ K_{\text{np1}} = F_K(SL)QK_{\text{basic}},\ K_{\text{pn0}} = K_{\text{pn1}} = K_{\text{basic}}\gamma^2 \quad \text{(S1.5)}$$

Here, $\mu > 1$ controls the degree of cross-bridge inhibition for the T/T units in states other than Ca-on, and Q controls the ratio of binding states for the myosin heads. The greater the value of Q, the larger the ratio of the binding states for a given Ca^{2+} concentration. In this study, $Q = 2.0$ was used for the DSE mode, while $Q = 2.2$ was used in the BSE model to make the maximal force same as that of the DSE model. To reproduce the SL ($SL = SL_0\lambda$) dependence in the active contraction tension, the following function $F_K(SL)$ was multiplied to define K_{np0} and K_{np1}.

$$F_K(SL) = \begin{cases} 1, & SL \geq SL_Q, \\ 1 - \alpha_Q(SL_Q - SL), & SL < SL_Q. \end{cases} \quad \text{(S1.6)}$$

The values α_Q = 1[1 / μm] and SL_Q = 2.2 μm were used in this study.

The rate constants of attachment f_a and detachment f_b were found based on the assumed free energies E_{wb} and E_0 of the P_{XB} state and the XB_{PreR} state, respectively:

$$f_a = c_{\text{app}} \exp\left(-\frac{E_0 - E_{wb}}{k_B T}\right), \quad f_b = c_{\text{app}}. \quad \text{(S1.7)}$$

The initial rod strain during attachment was given stochastically based on a Boltzmann distribution determined from the rod strain energy $W(x)$ [19]. The detachment rate-constant for the transitions from the XB_{PostR2} state to the N_{XB} state and from the N_{ATP} state to the N_{XB} state were g_{NXB} and g_{ATP} respectively, both listed in Table S1.

We also considered forced detachments caused by the extreme strain of a myosin rod using the rate constants:

$$d_{\text{PostR}i}(x) = \begin{cases} 0, & x < x_{\text{for}}, \\ c_{\text{for}}\left(\exp\left(a_{\text{for}}(x - x_{\text{for}})\right) - 1\right), & x \geq x_{\text{for}}, \end{cases} \quad i = 1, 2. \quad \text{(S1.8)}$$

The values of a_{for} = 0.05 [1 / nm] and x_{for} = 9 nm were adopted for this study. c_{for} is listed in Table S1. Here, we assumed that forced detachment from the XB_{PreR} state led to the P_{XB} state, while forced detachment from the XB_{PostR1} or the XB_{PostR2} states led to the N_{ATP} state.

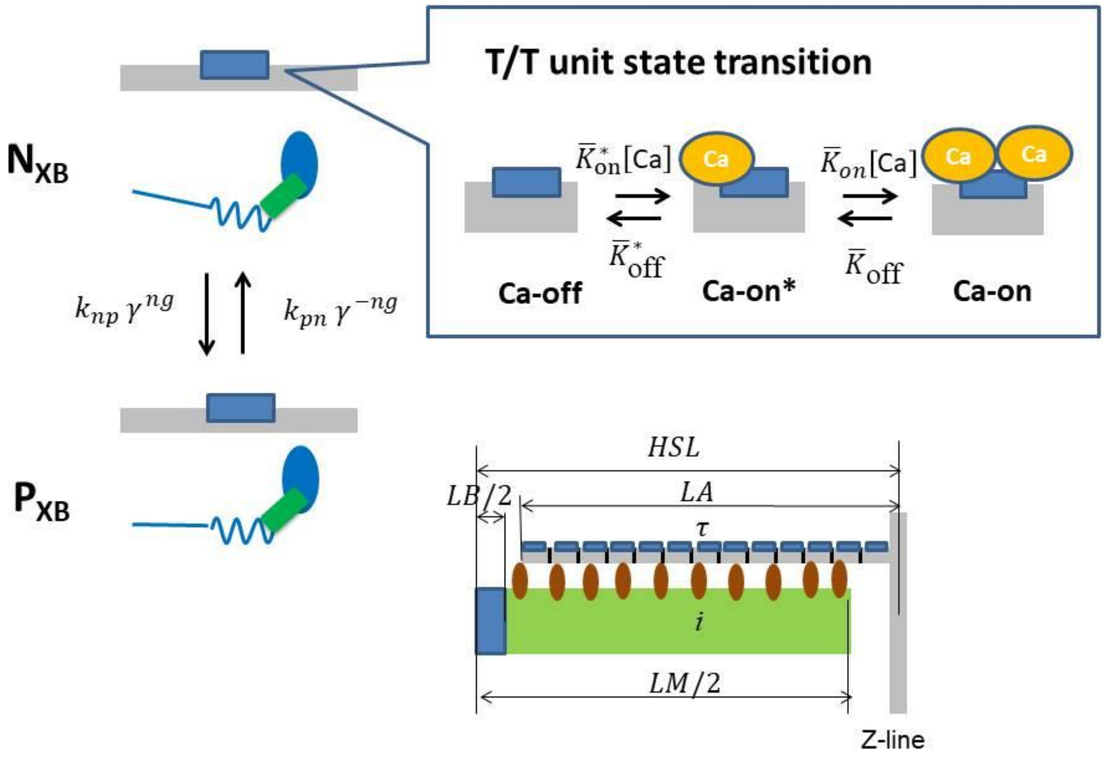

Figure S1.1 Transitions of the myosin states (left), transitions of the T/T unit states (right), and geometry of the half-sarcomere model (right-bottom).

S1.2 The strain energy for the myosin rod

The elastic force of a myosin rod is nonlinear with respect to the strain, as reported by Kaya et al. [5]. We assumed that the myosin rods behaved as linear springs for positive stretches, whereas nonlinear behaviour was introduced for negative stretches because of the slack induced along the myosin rod. The strain energy W was found by integrating the force F_{rod} from $x = 0$ defined by:

$$F_{rod}(x) = \begin{cases} b_{xb}k_{xb}(x+\xi_1) - F_1, & x < -\xi_1, \\ \dfrac{k_{xb}}{a_{xb}}(\exp(a_{xb}x) - 1), & -\xi_1 \le x < 0, \\ k_{xb}x, & 0 \le x. \end{cases} \tag{S1.9}$$

where a_{xb} and F_1 are determined from the other parameters, so that the function F_{rod} and its first derivative are continuous at $\xi = 0$ and $-\xi_1$:

$$\begin{cases} a_{xb} = -\dfrac{(\ln b_{xb})}{\xi_1} \\ F_1 = \dfrac{k_{xb}(1 - \exp(-a_{xb}\xi_1))}{a_{xb}} \end{cases} \tag{S1.10}$$

Table S1.1 Parameters for the actomyosin dynamics. 'adjusted' indicates that they were adjusted to reproduce the phenomena.

Parameter	Value	Unit	Reference	Parameter	Value	Unit	Reference
**ATP hydrolysis energy**				**Sarcomere Geometry**			
E_{ATP}	76.5	$\mathrm{pN} \cdot \mathrm{nm}$	[6]	SL_0	1.9	μm	[3]
$k_B T$	4.28	$\mathrm{pN} \cdot \mathrm{nm}$	$T = 310$	LM	1.65	μm	[2]
**Stroke sizes and Free energies**				LB	0.16	μm	[1]
s_1 , s_2	5.5	nm	[6]	LA	1.0	μm	[1]
E_{wb}	E_{ATP}	pN/nm	[6]	SA_0	693	nm^2	[7]
E_0	$1.05E_{wb}$	pN/nm	[6]	**Force regulation through T/T-unit**			
E_1	$0.82E_{wb}$	pN/nm	[6]	K_{on}^*	$150R_\mathbf{0}$	1/s	adjusted
E_2	0	pN/nm	[6]	K_{off}^*	80	1/s	adjusted
				${K'}_{\mathrm{on}}^*$	150	1/s	adjusted
				${K'}_{\mathrm{off}}^*$	20	1/s	adjusted
**Rod strain energy *W***				K_{on}	$150R_\mathbf{0}$	1/s	adjusted
k_{xb}	2.0	pN/nm	[6]	K_{off}	$80R_\mathbf{0}$	1/s	adjusted
b_{xb}	0.05	unitless	[6]	${K'}_{\mathrm{on}}$	150	1/s	adjusted
ξ_1	4.35	nm	[6]	${K'}_{\mathrm{off}}$	40	1/s	adjusted
				K_{basic}	20	1/s	[6]
				Q	2.0	unitless	[6]
**Power-stroke transitions**				μ	15	unitless	[6]
C_{T}	0.55	unitless	adjusted				
h_1	$1700C_{\mathrm{T}}$	1/s	adjusted	γ	40	unitless	[3]
h_2	$1700C_{\mathrm{T}}$	1/s	adjusted				
				**Number of elements in a sarcomere**			
**Detachment transitions**				N_M	38	unitless	[6]
c_{app}	$5250C_{\mathrm{T}}$	1/s	[19]	N_T	32	unitless	[6]
g_{ATP}	$10000C_{\mathrm{T}}$	1/s		N_F	16	unitless	adjusted
g_{NXB}	$225C_{\mathrm{T}}$	1/s	[19]				
c_{for}	$20C_{\mathrm{T}}$	1/s	adjusted				

References for S1

[1] Kolb, J., Li, F., Methawasin, M., Adler, M., Escobar, Y. N., Nedrud, J., Pappas, C. T., Harris, S. P. & Granzier, H., Thin filament length in the cardiac sarcomere varies with sarcomere length but is independent of titin and nebulin, J Mol Cell Cardiol. **97**, 286–294 (2016).

[2] Lodish, H., Berk, A., Zipursky, S. L., Matsudaira, P., Baltimore, D. & Darnell, J., Molecular Cell Biology, 4th edition, New York: W. H. Freeman (2000).

[3] Rice, J. J., Wang, F., Bers, D. M. & de Tombe, P. P., Approximate model of cooperative activation and crossbridge cycling in cardiac muscle using ordinary differential equations, Biophys J. **95**(5), 2368–2390 (2008).

[4] Rodriguez, E. K., Omens, J. H., Waldman, L. K. & McCulloch, A. D., Effect of residual stress on transmural sarcomere length distributions in rat left ventricle, Am J Physiol. **264**(4 Pt 2), H1048–1056 (1993).

[5] Kaya, M., & Higuchi, H.. Non-linear elasticity and an 8 nm working stroke of single myosin molecules in myofilaments, Science **329**(5992), 686–689 (2010).

[6] Washio, T., Yoneda, K., Okada, J., Kariya, T., Sugiura, S. & Hisada, T., Ventricular fiber optimization utilizing the branching structure, Int. J. Numer. Method Biomed. Eng. **32**:e02753 (2016).

[7] Sato, K., Kuramoto, Y., Ohtaki, M., Shimamoto, Y. & Ishiwata, S. Locally and globally coupled oscillators in muscle. Phys Rev Lett. **111**(10), 108104 (2013).

S2 FE ventricular model

S2.1 Passive and Viscous Parts of the Ventricle Model

Here, the parameters used in this study are listed in Table S2.1. The passive stress tensor $\boldsymbol{S}_{\mathrm{pas}}$ in Equation (29) is given by the deformation potential function W_{pas} as

$$\boldsymbol{S}_{\mathrm{pas}} = \frac{\partial W_{\mathrm{pas}}}{\partial \boldsymbol{E}}^{\mathrm{T}} \quad (S2.1)$$

The potential function W_{pas} is determined by a macroscopic passive potential:

$$W_{\mathrm{pas}} = c_1(\tilde{I}_1 - 3) + c_u \frac{\exp(Q_u) - 1}{2} \quad (S2.2)$$

where $\tilde{I}_1$ is the reduced invariant defined as

$$\tilde{I}_1 = \det(\boldsymbol{C})^{-\frac{1}{3}} \mathrm{Tr}(\boldsymbol{C}) \quad (S2.3)$$

with the right Cauchy-Green deformation tensor $\boldsymbol{C} = \boldsymbol{F}^{\mathrm{T}}\boldsymbol{F}$. Q_u is a quadratic form of the Green-Lagrange strain tensor[1]:

$$Q_u = b_{ff}E_{ff}^2 + b_{ss}E_{ss}^2 + b_{nn}E_{nn}^2 + 2b_{fs}E_{fs}^2 + 2b_{fn}E_{fn}^2 + 2b_{sn}E_{sn}^2 \quad (S2.4)$$

where the components are defined based on the fiber-sheet structure of the muscle walls as

$$\begin{cases} E_{ff} = \boldsymbol{E}:\boldsymbol{f} \otimes \boldsymbol{f}, E_{ss} = \boldsymbol{E}:\boldsymbol{s} \otimes \boldsymbol{s},\ E_{nn} = \boldsymbol{E}:\boldsymbol{n} \otimes \boldsymbol{n} \\ E_{fs} = \boldsymbol{E}:\boldsymbol{f} \otimes \boldsymbol{s}, E_{fn} = \boldsymbol{E}:\boldsymbol{f} \otimes \boldsymbol{n}, E_{sn} = \boldsymbol{E}:\boldsymbol{s} \otimes \boldsymbol{n} \end{cases} \quad (S2.5)$$

Here, $\{\boldsymbol{f}, \boldsymbol{s}, \boldsymbol{n}\}$ is the orthonormal basis of the ventricle walls that determines the fiber and laminar structures.

For the viscous part, the Newtonian viscosity $\boldsymbol{S}_{\mathrm{vis}}$ in Equation (29) is given by

$$\boldsymbol{S}_{\mathrm{vis}} = 2\mu_S J \boldsymbol{F}^{-1}\boldsymbol{D}_S(\boldsymbol{F}^{-1})^{\mathrm{T}} \quad (S2.6)$$

where μ_S is the viscosity coefficient, and $\boldsymbol{D}_S$ is the deformation velocity tensor defined as

$$\boldsymbol{D}_S = \frac{1}{2}\left(\frac{\partial \dot{\boldsymbol{u}}}{\partial \boldsymbol{x}} + \frac{\partial \dot{\boldsymbol{u}}}{\partial \boldsymbol{x}}^T\right) \quad (S2.7)$$

Note that the derivatives are given with respect to the Eulerian coordinates $\boldsymbol{x}$.

Table S2.1 Parameters for the muscle material properties in the biventricular model.

Parameter	Value	Unit	Parameter	Value	Unit
**Muscle Passive**			**Sarcomere**		
c_1	71.8	Pa	SA_0	0.001	μm^2
c_u	1000	Pa	R_S	0.5	unitless
b_{ff}	3	unitless			
b_{ss}	7	unitless			
b_{nn}	3	unitless			
b_{fs}	12	unitless			
b_{fn}	3	unitless			
b_{sn}	3	unitless			
μ_V	36.66	$\mathrm{Pa \cdot s}$			
κ	800	KPa			

S2.2. Circulatory System

The ventricle blood pressures P_{L} and P_{R} in Equation (23) were determined through their interactions with the circulatory system of the body. These were modeled as electrical analog circuit. In particular, the flow rates at the inlets and the outlets were associated with the rates of volume change in the cavity according to Equations (25, 26) where F_{MI}, F_{AO}, F_{TR} and F_{PA} were the flow rates, respectively, through the mitral, aortic, tricuspid, and pulmonary valves (Figure S2). These flow rates were determined by Ohm's law while taking the rectification of the valve into account.

$$F = H(\bar{F})\bar{F} \tag{S2.8}$$

Here, $\bar{F}$ was the flow rate in the case of no rectification, and H was the relaxed Heaviside function:

$$H(\bar{F}) = \begin{cases} 0, & \bar{F} < 0, \\ \left(\frac{\bar{F}}{\bar{F}_0}\right)^2 \left(3 - 2\frac{\bar{F}}{\bar{F}_0}\right), & 0 \leq \bar{F} \leq \bar{F}_0, \\ 1, & \bar{F}_0 < \bar{F} \end{cases} \tag{S2.9}$$

In our simulation, the value $\bar{F}_0 = 5\ \mathrm{ml/L}$ was used.

As the atrial model, the formulations by Kaye et al.[2] were applied. The left and right atrial pressure P_{A} is related to the time-varying elasticity e_{A} and the chamber volume V_{A} as follows. Hereafter, the subscript "A" stands for "LA" or "RA" for the left or the right atrium, respectively.

$$P_{\mathrm{A}}(e_{\mathrm{A}}, V_{\mathrm{A}}) = P_{\mathrm{A,ed}}(V_{\mathrm{A}}) + e_{\mathrm{A}}\left(P_{\mathrm{A,es}}(V_{\mathrm{A}}) - P_{\mathrm{A,ed}}(V_{\mathrm{A}})\right) \tag{S2.10}$$

Here, the functions $P_{\mathrm{A,ed}}$ and $P_{\mathrm{A,es}}$ give, respectively, the pressure at the end of the diastolic and systolic phases for chamber volume V_{A}. These functions are defined by

$$\begin{cases} P_{\mathrm{A,ed}}(V_{\mathrm{A}}) = \beta_{\mathrm{A}}\left(\exp\left(\alpha_{\mathrm{A}}\left(V_{\mathrm{A}} - V_{\mathrm{A,0}}\right)\right) - 1\right) \\ P_{\mathrm{A,es}}(V_{\mathrm{A}}) = E_{\mathrm{A,es}}\left(V_{\mathrm{A}} - V_{\mathrm{A,0}}\right) \end{cases} \tag{S2.11}$$

The time-varying elastance e_{A} is given as a function of the time T as follows:

$$e_{\mathrm{A}}(T) = \begin{cases} \frac{1}{2}\left(\sin\pi\left(\frac{T - T_0}{T_{\mathrm{max}}} - \frac{1}{2}\right) + 1\right), & T \le \frac{3}{2}T_{\mathrm{max}} + T_0 \\ \frac{1}{2}\exp\left(-\frac{\left(T - T_0 - \frac{3}{2}T_{\mathrm{max}}\right)}{\tau_A}\right), & T > \frac{3}{2}T_{\mathrm{max}} + T_0 \end{cases} \tag{S2.12}$$

Here, T_0 is the start time of atrial excitation, T_{max} is the time to maximal chamber elastance, and τ_{A} is the time constant of relaxation. Together with the other part of the circuit model of Figure S2.1, the overall equations (Equation (28) in the main text) for pulmonary circulation are given by

$$\begin{cases} F_{\mathrm{PA}} - H(\bar{F}_{\mathrm{PA}})\bar{F}_{\mathrm{PA}} = 0 \\ \dot{Q}_{\mathrm{AP}} + F_{\mathrm{PA}} + \frac{1}{R_{\mathrm{AP}}}\left(\frac{Q_{\mathrm{AP}}}{C_{\mathrm{AP}}} - \frac{Q_{\mathrm{VP}}}{C_{\mathrm{VP}}}\right) = 0 \\ \dot{Q}_{\mathrm{PV}} - \frac{1}{R_{\mathrm{AP}}}\left(\frac{Q_{\mathrm{AP}}}{C_{\mathrm{AP}}} - \frac{Q_{\mathrm{VP}}}{C_{\mathrm{VP}}}\right) + \frac{1}{R_{\mathrm{VP}}}\left(\frac{Q_{\mathrm{VP}}}{C_{\mathrm{VP}}} - P_{\mathrm{LA}}(e_{\mathrm{LA}}, V_{\mathrm{LA}})\right) = 0 \\ F_{\mathrm{MI}} - H(\bar{F}_{\mathrm{MI}})\bar{F}_{\mathrm{MI}} = 0 \\ \dot{V}_{\mathrm{LA}} - \frac{1}{R_{\mathrm{VP}}}\left(\frac{Q_{\mathrm{VP}}}{C_{\mathrm{VP}}} - P_{\mathrm{LA}}(e_{\mathrm{LA}}, V_{\mathrm{LA}})\right) - F_{\mathrm{MI}} = 0 \end{cases} \tag{S2.13}$$

Here, C denotes the pulmonary venous compliance, and Q denotes the increase of the blood volume from zero pressure. $\bar{F}_{\mathrm{PA}}$ and $\bar{F}_{\mathrm{MI}}$, the flow rates in the case of no rectification, are given by

$$\begin{cases} \bar{F}_{\mathrm{PA}} = \frac{1}{R_{\mathrm{PA}}}\left(P_{\mathrm{R}} - \frac{Q_{\mathrm{AP}}}{C_{\mathrm{AP}}}\right) \\ \bar{F}_{\mathrm{MI}} = \frac{1}{R_{\mathrm{LA}}}\left(P_{\mathrm{LA}}(e_{\mathrm{LA}}, V_{\mathrm{LA}}) - P_{\mathrm{L}}\right) \end{cases} \tag{S2.14}$$

Similarly, the overall equations (Equation (27) in the main text) for the systemic circulation are given by

$$\begin{cases} F_{\mathrm{AO}} - H(\bar{F}_{\mathrm{AO}})\bar{F}_{\mathrm{AO}} = 0 \\ \dot{Q}_{\mathrm{A}} + F_{\mathrm{AO}} + \dfrac{1}{R_{\mathrm{A}}}\left(\dfrac{Q_{\mathrm{A}}}{C_{\mathrm{A}}} - \dfrac{Q_{\mathrm{VS}}}{C_{\mathrm{VS}}}\right) = 0 \\ \dot{Q}_{\mathrm{VS}} - \dfrac{1}{R_{\mathrm{A}}}\left(\dfrac{Q_{\mathrm{A}}}{C_{\mathrm{A}}} - \dfrac{Q_{\mathrm{VS}}}{C_{\mathrm{VS}}}\right) + \dfrac{1}{R_{\mathrm{VS}}}\left(\dfrac{Q_{\mathrm{VS}}}{C_{\mathrm{VS}}} - P_{\mathrm{RA}}(e_{\mathrm{RA}}, V_{\mathrm{RA}})\right) = 0 \\ F_{\mathrm{TR}} - H(\bar{F}_{\mathrm{TR}})\bar{F}_{\mathrm{TR}} = 0 \\ \dot{V}_{\mathrm{RA}} - \dfrac{1}{R_{\mathrm{VS}}}\left(\dfrac{Q_{\mathrm{VS}}}{C_{\mathrm{VS}}} - P_{\mathrm{RA}}(e_{\mathrm{RA}}, V_{\mathrm{RA}})\right) - F_{\mathrm{TR}} = 0 \end{cases} \tag{S2.15}$$

with flow rates (for no rectification)

$$\begin{cases} \bar{F}_{\mathrm{AO}} = \dfrac{1}{R_{\mathrm{C}}}\left(P_{\mathrm{L}} - \dfrac{Q_{\mathrm{A}}}{C_{\mathrm{A}}}\right) \\ \bar{F}_{\mathrm{TR}} = \dfrac{1}{R_{\mathrm{RA}}}\left(P_{\mathrm{RA}}(e_{\mathrm{RA}}, V_{\mathrm{RA}}) - P_{\mathrm{R}}\right) \end{cases} \tag{S2.16}$$

The parameters adopted in our simulation are listed in Table S2.2. The parameter values were chosen to reproduce the temporal changes in ventricular blood pressure for a standard healthy heart.

Table S2.2 Parameters for the pulmonary and systemic circulations, with T_{cycle} standing for the period of a heartbeat

Parameter	Value	Unit	Parameter	Value	Unit
**Pulmonary Circulation**			**Systemic Circulation**		
C_{AP}	6.56	$\mathrm{ml} \cdot \mathrm{mmHg}^{-1}$	C_{A}	2.61	$\mathrm{ml} \cdot \mathrm{mmHg}^{-1}$
C_{VP}	12.17	$\mathrm{ml} \cdot \mathrm{mmHg}^{-1}$	C_{VS}	54.9	$\mathrm{ml} \cdot \mathrm{mmHg}^{-1}$
R_{PA}	0.014	$\mathrm{mmHg} \cdot \mathrm{s} \cdot \mathrm{ml}^{-1}$	R_{C}	0.0512	$\mathrm{mmHg} \cdot \mathrm{s}$
R_{AP}	0.117	$\mathrm{mmHg} \cdot \mathrm{s} \cdot \mathrm{ml}^{-1}$	R_{A}	1.08	$\mathrm{mmHg} \cdot \mathrm{s}$
R_{VP}	0.0025	$\mathrm{mmHg} \cdot \mathrm{s} \cdot \mathrm{ml}^{-1}$	R_{VS}	0.068	$\mathrm{mmHg} \cdot \mathrm{s}$
R_{LA}	0.025	$\mathrm{mmHg} \cdot \mathrm{s} \cdot \mathrm{ml}^{-1}$	R_{RA}	0.025	$\mathrm{mmHg} \cdot \mathrm{s}$
**Left Atrium**			**Right Atrium**		
α_{LA}	0.044	ml^{-1}	α_{RA}	0.044	ml^{-1}
β_{LA}	0.3	mmHg	β_{RA}	0.3	mmHg
$E_{\mathrm{LA,es}}$	0.3	$\mathrm{mmHg} \cdot \mathrm{ml}^{-1}$	$E_{\mathrm{RA,es}}$	0.25	$\mathrm{mmHg} \cdot \mathrm{ml}^{-1}$
$V_{\mathrm{LA,0}}$	5.0	ml	$V_{\mathrm{RA,0}}$	5.0	ml
τ_{LA}	0.025	s	τ_{RA}	0.025	s
T_{max}	0.125	s	T_{max}	0.125	s
$T_{\mathrm{cycle}} - T_0$	0.15	s	$T_{\mathrm{cycle}} - T_0$	0.15	s

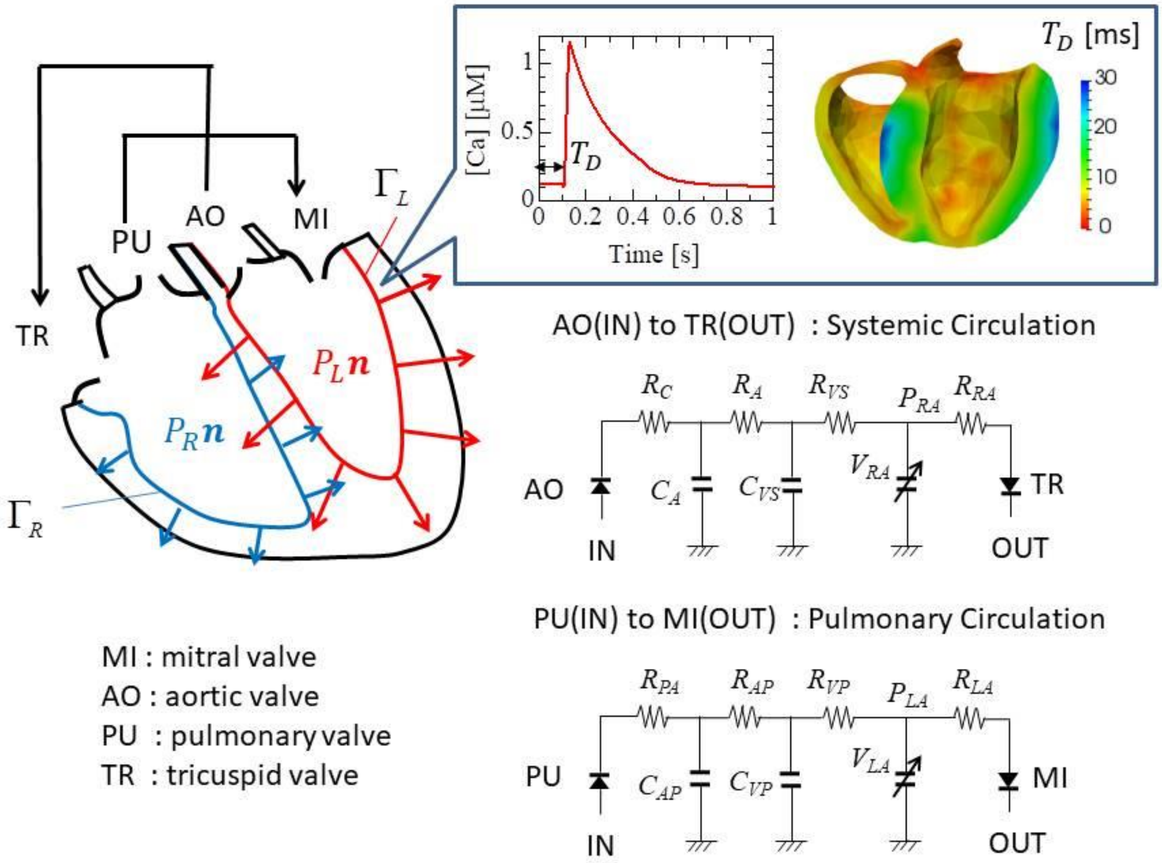

Figure S2.1 Electrical analog circuits connected to the valve interfaces. The systemic circulation model represents blood flow from the left to the right ventricle through the aortic (AO) and the tricuspid (TR) valves, while the pulmonary circulation model represents blood flow from the right to the left ventricle through the pulmonary (PU) and the mitral (MI) valves. The boxed inset shows the Ca^{2+}-transient profile and the transmural delay of the rise of $[Ca^{2+}]$.

References for S2

[1] Usyk, T. P., Mazhari, R., and McCulloch, A. D. (2000). Effect of laminar orthotrophic myofiber architecture on regional stress and strain in the canine left ventricle. Journal of Elasticity 61,143–164.

[2] Kaye, D., Shah, S. J., Borlaug, B. A., Gustafsson, F., Komtebedde, J., Kubo, S., Magnin, C., Maurer, M. S., Feldman, T., and Burkhoff, D. (2014). Effects of an interatrial shunt on rest and exercise hemodynamics: results of a computer simulation in heart failure. J Card Fail. 20, 212–221.

S3 Relationship of active tension, stretch rate, and ATP consumption rate

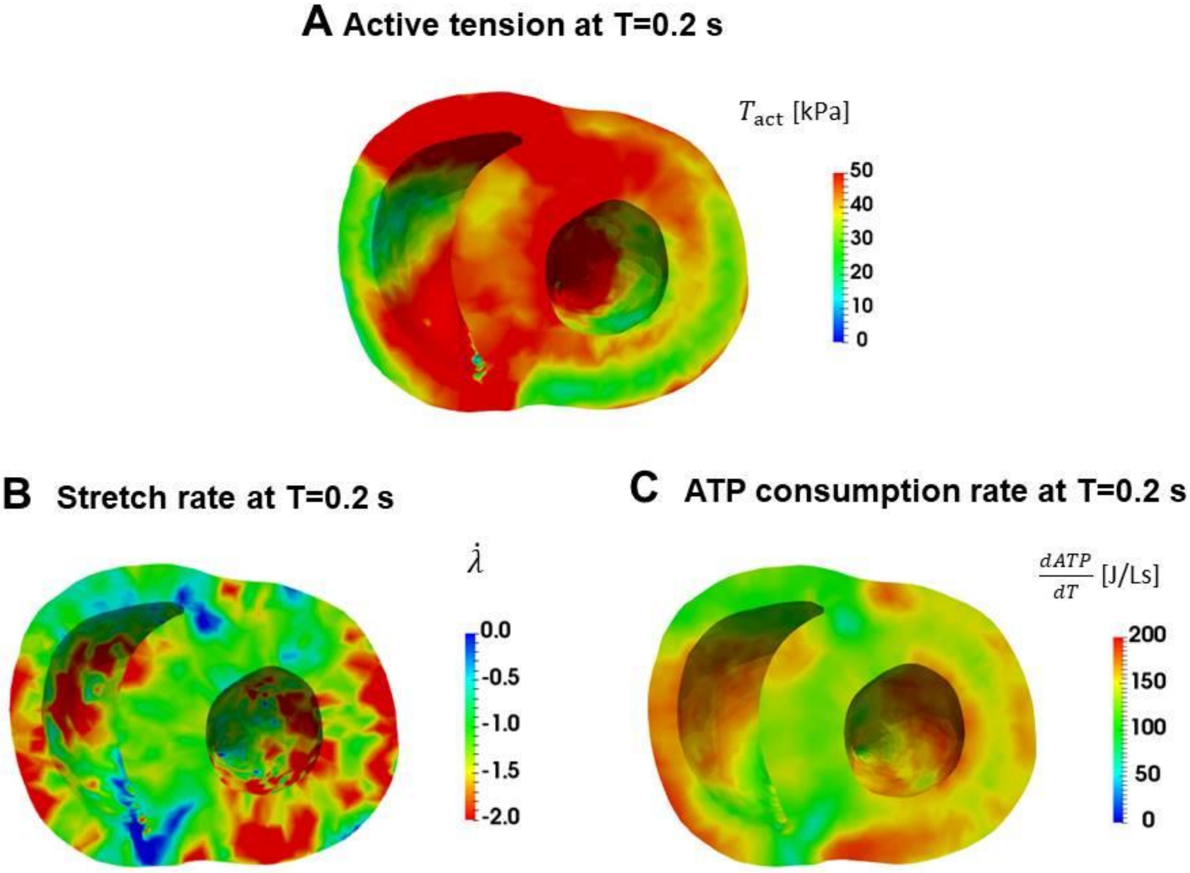

Figure S3.1 Distributions of active tension (A), stretch rate (B), and ATP consumption rate (C) at T = 0.2 s in the contraction phase in the middle cross-section perpendicular to the long axis. These values were averaged on the nodes in the FE mesh. The results were obtained with N_F =64.
